# Supplementary figures and images for: Determination of variable region sequences from hybridoma immunoglobulins that target Mycobacterium tuberculosis virulence factors
Source: PLoS One. 2021 Aug 20;16(8):e0256079. doi: 10.1371/journal.pone.0256079 (PMC8378720; doi:10.1371/journal.pone.0256079)

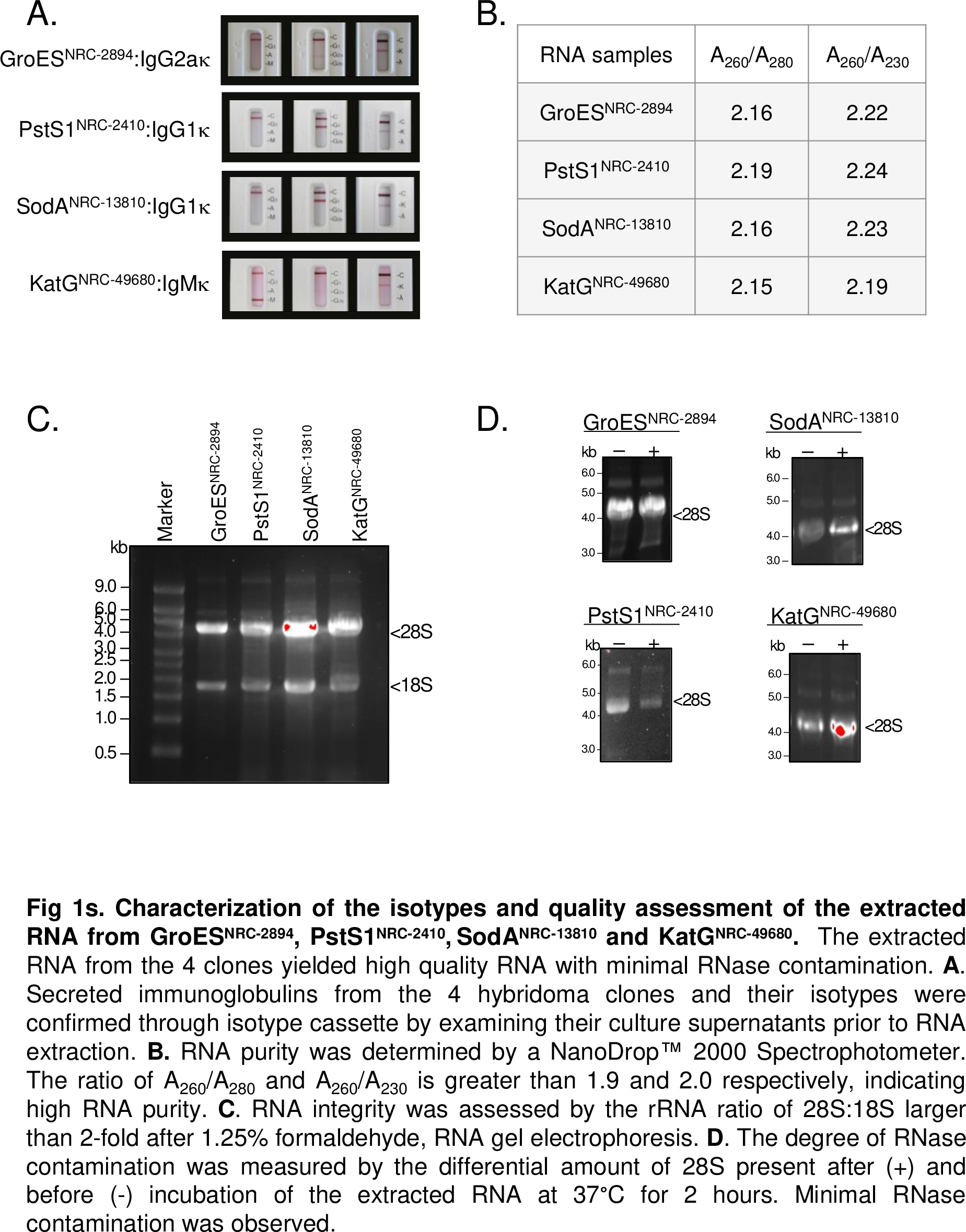

Supplement: S1 Fig — The extracted RNA from the 4 clones yielded high quality RNA with minimal RNase contamination. A. Secreted immunoglobulins from the 4 hybridoma clones and their isotypes were confirmed through isotype cassette by examining their culture supernatants prior to RNA extraction. B. RNA purity was determined by a NanoDrop™ 2000 Spectrophotometer. The ratio of A260/A280 and A260/A230 is greater than 1.9 and 2.0 respectively, indicating high RNA purity. C. RNA integrity was assessed by the rRNA ratio of 28S:18S larger than 2-fold after 1.25% formaldehyde, RNA gel electrophoresis. D. The degree of RNase contamination was measured by the differential amount of 28S present after (+) and before (-) incubation of the extracted RNA at 37°C for 2 hours. Minimal RNase contamination was observed. (TIF) [file pone.0256079.s001.tif]

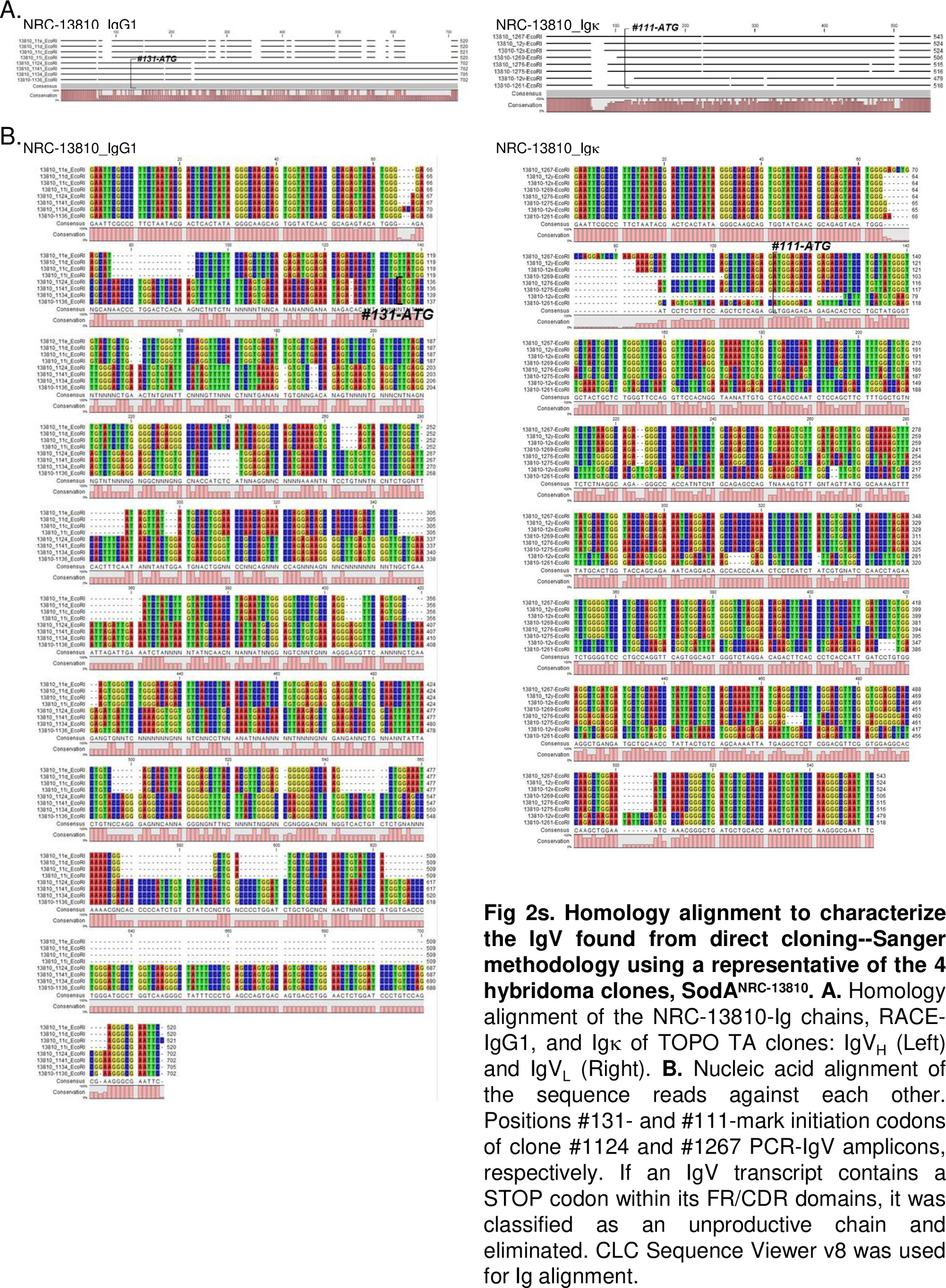

Supplement: S2 Fig — A. Homology alignment of the NRC-13810-Ig chains, RACE-IgG1, and Igκ of TOPO TA clones: IgVH (Left) and IgVL (Right). B. Nucleic acid alignment of the sequence reads against each other. Positions #131- and #111-mark initiation codons of clone #1124 and #1267 PCR-IgV amplicons, respectively. If an IgV transcript contains a STOP codon within its FR/CDR domains, it was classified as an unproductive chain and eliminated. CLC Sequence Viewer v8 was used for Ig alignment. (TIF) [file pone.0256079.s002.tif]

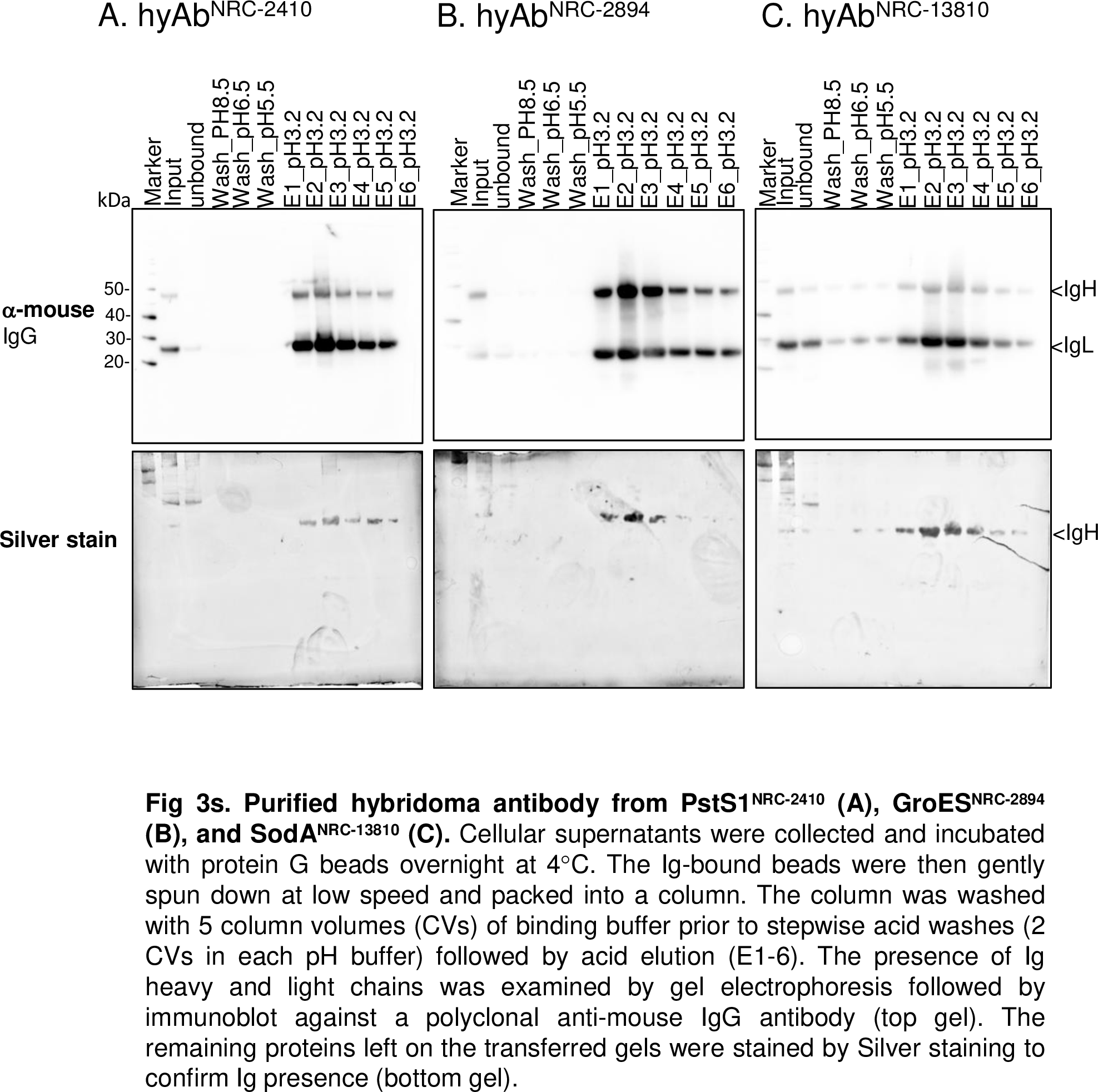

Supplement: S3 Fig — Purified hybridoma antibody from PstS1NRC-2410 (A), GroESNRC-2894 (B), and SodANRC-13810 (C). Cellular supernatants were collected and incubated with protein G beads overnight at 4°C. The Ig-bound beads were then gently spun down at low speed and packed into a column. The column was washed with 5 column volumes (CVs) of binding buffer prior to stepwise acid washes (2 CVs in each pH buffer) followed by acid elution (E1-6). The presence of Ig heavy and light chains was examined by gel electrophoresis followed by immunoblot against a polyclonal anti-mouse IgG antibody (top gel). The remaining proteins left on the transferred gels were stained by Silver staining to confirm Ig presence (bottom gel). (TIF) [file pone.0256079.s003.tif]

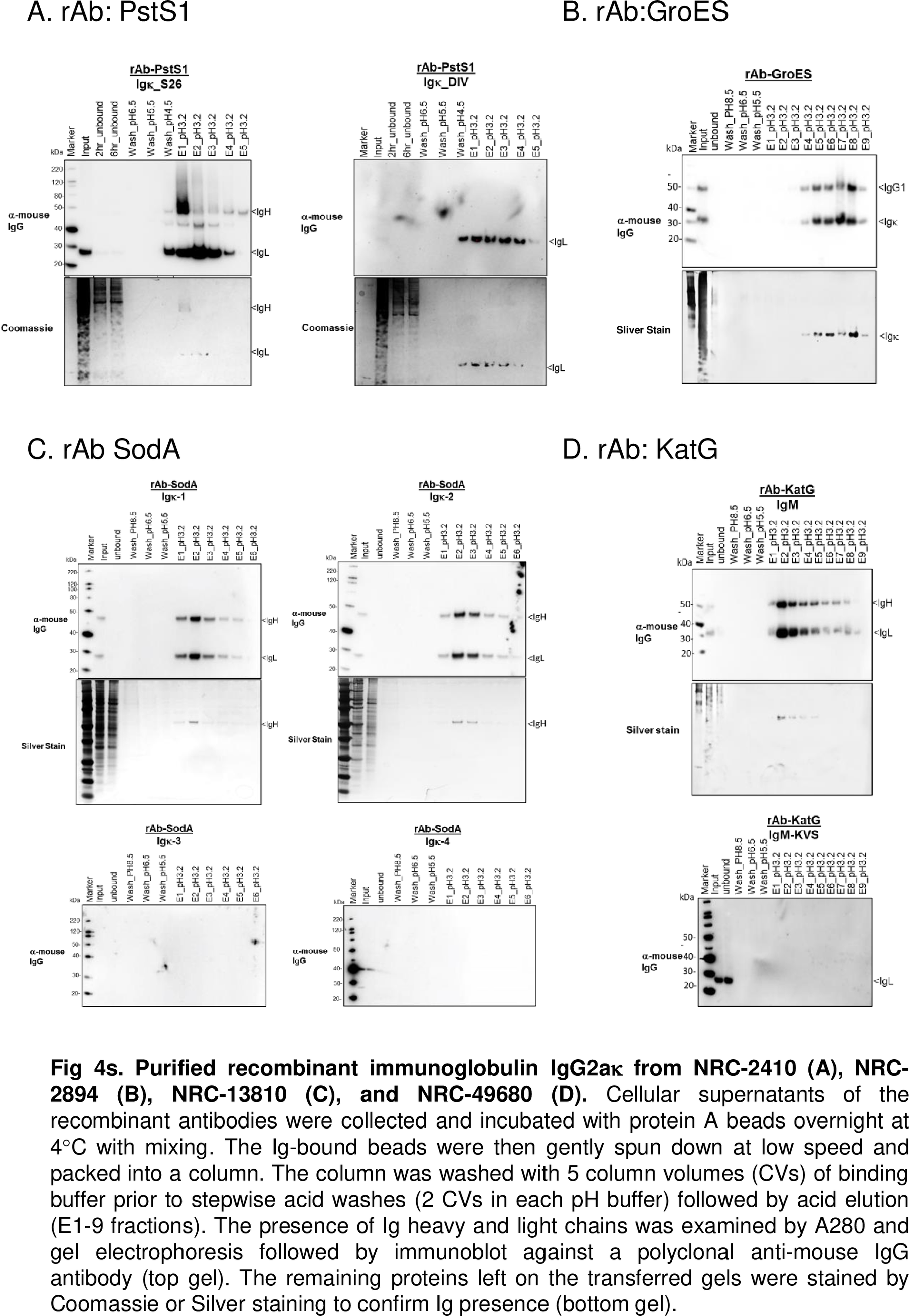

Supplement: S4 Fig — Purified recombinant immunoglobulin IgG2aκ from NRC-2410 (A), NRC-2894 (B), NRC-13810 (C), and NRC-49680 (D). Cellular supernatants of the recombinant antibodies were collected and incubated with protein A beads overnight at 4°C with mixing. The Ig-bound beads were then gently spun down at low speed and packed into a column. The column was washed with 5 column volumes (CVs) of binding buffer prior to stepwise acid washes (2 CVs in each pH buffer) followed by acid elution (E1-9 fractions). The presence of Ig heavy and light chains was examined by A280 and gel electrophoresis followed by immunoblot against a polyclonal anti-mouse IgG antibody (top gel). The remaining proteins left on the transferred gels were stained by Coomassie or Silver staining to confirm Ig presence (bottom gel). (TIF) [file pone.0256079.s004.tif]

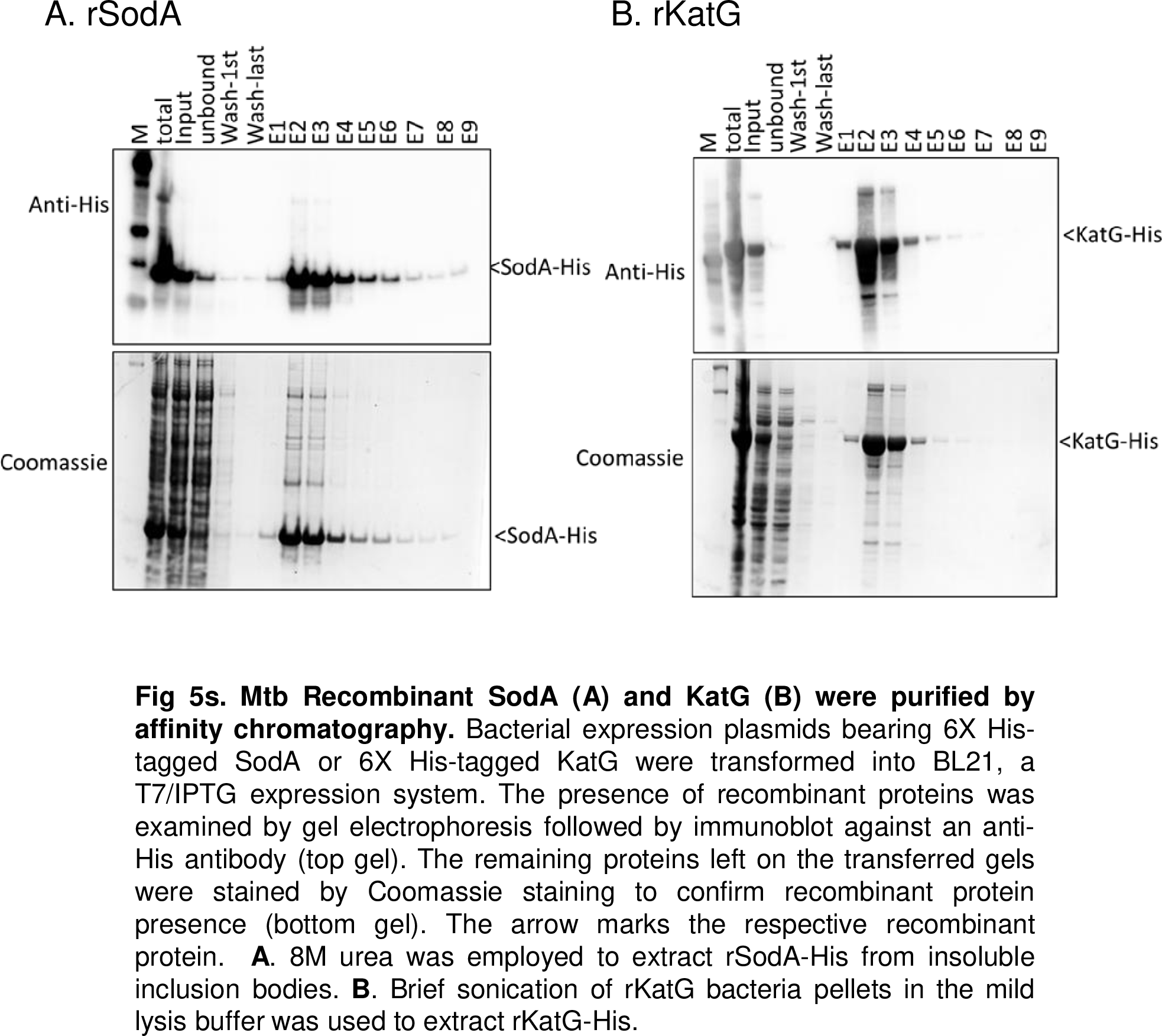

Supplement: S5 Fig — Mtb Recombinant SodA (A) and KatG (B) were purified by affinity chromatography. Bacterial expression plasmids bearing 6X His-tagged SodA or 6X His-tagged KatG were transformed into BL21, a T7/IPTG expression system. The presence of recombinant proteins was examined by gel electrophoresis followed by immunoblot against an anti-His antibody (top gel). The remaining proteins left on the transferred gels were stained by Coomassie staining to confirm recombinant protein presence (bottom gel). The arrow marks the respective recombinant protein. A. 8M urea was employed to extract rSodA-His from insoluble inclusion bodies. B. Brief sonication of rKatG bacteria pellets in the mild lysis buffer was used to extract rKatG-His. (TIF) [file pone.0256079.s005.tif]

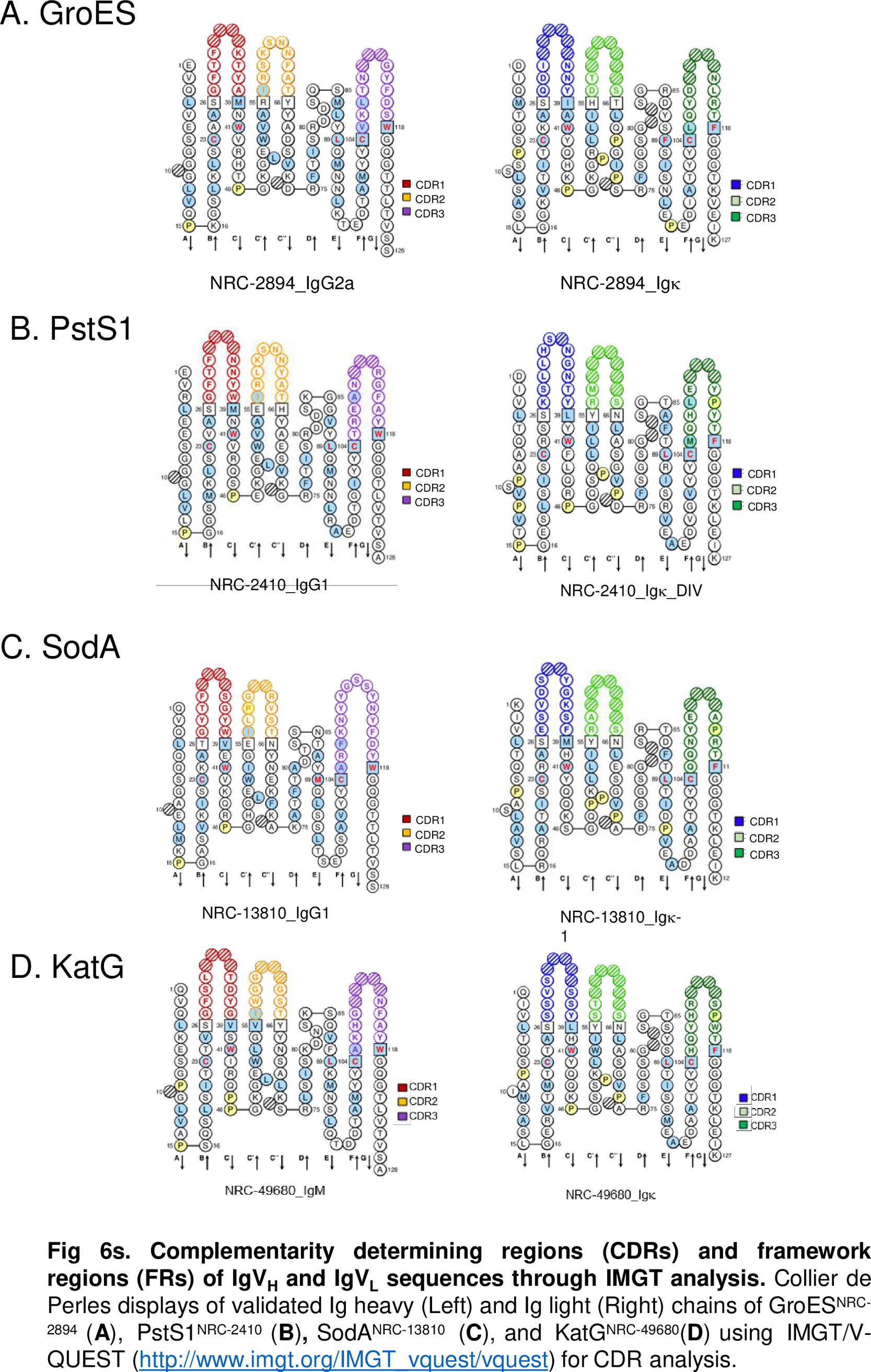

Supplement: S6 Fig — Collier de Perles displays of validated Ig heavy (Left) and Ig light (Right) chains of GroESNRC-2894 (A), PstS1NRC-2410 (B), SodANRC-13810 (C), and KatGNRC-49680(D) using IMGT/V-QUEST (http://www.imgt.org/IMGT_vquest/vquest) for CDR analysis. (TIF) [file pone.0256079.s006.tif]

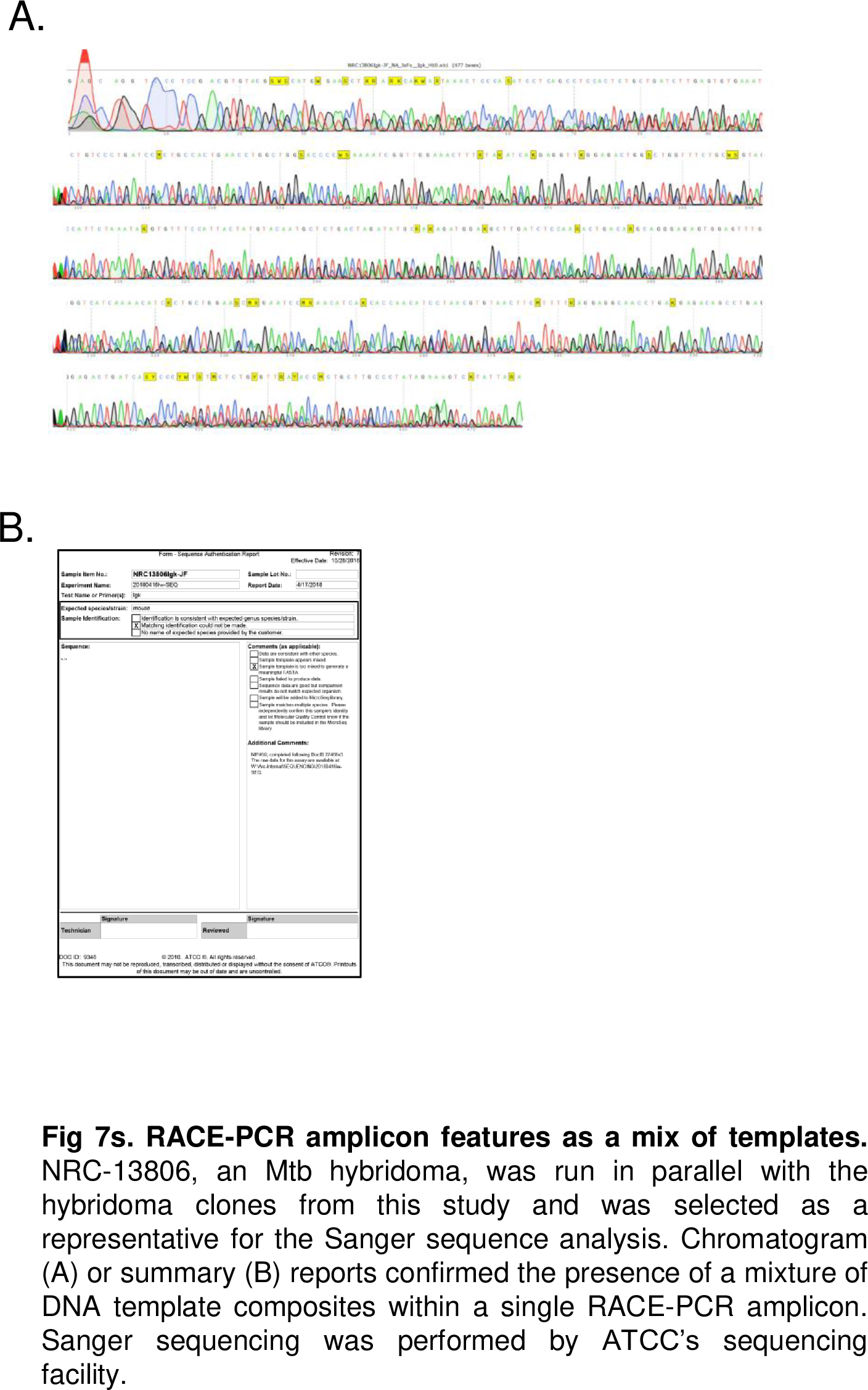

Supplement: S7 Fig — NRC-13806, an Mtb hybridoma, was run in parallel with the hybridoma clones from this study and was selected as a representative for the Sanger sequence analysis. Chromatogram (A) or summary (B) reports confirmed the presence of a mixture of DNA template composites within a single RACE-PCR amplicon. Sanger sequencing was performed by ATCC’s sequencing facility. (TIF) [file pone.0256079.s007.tif]

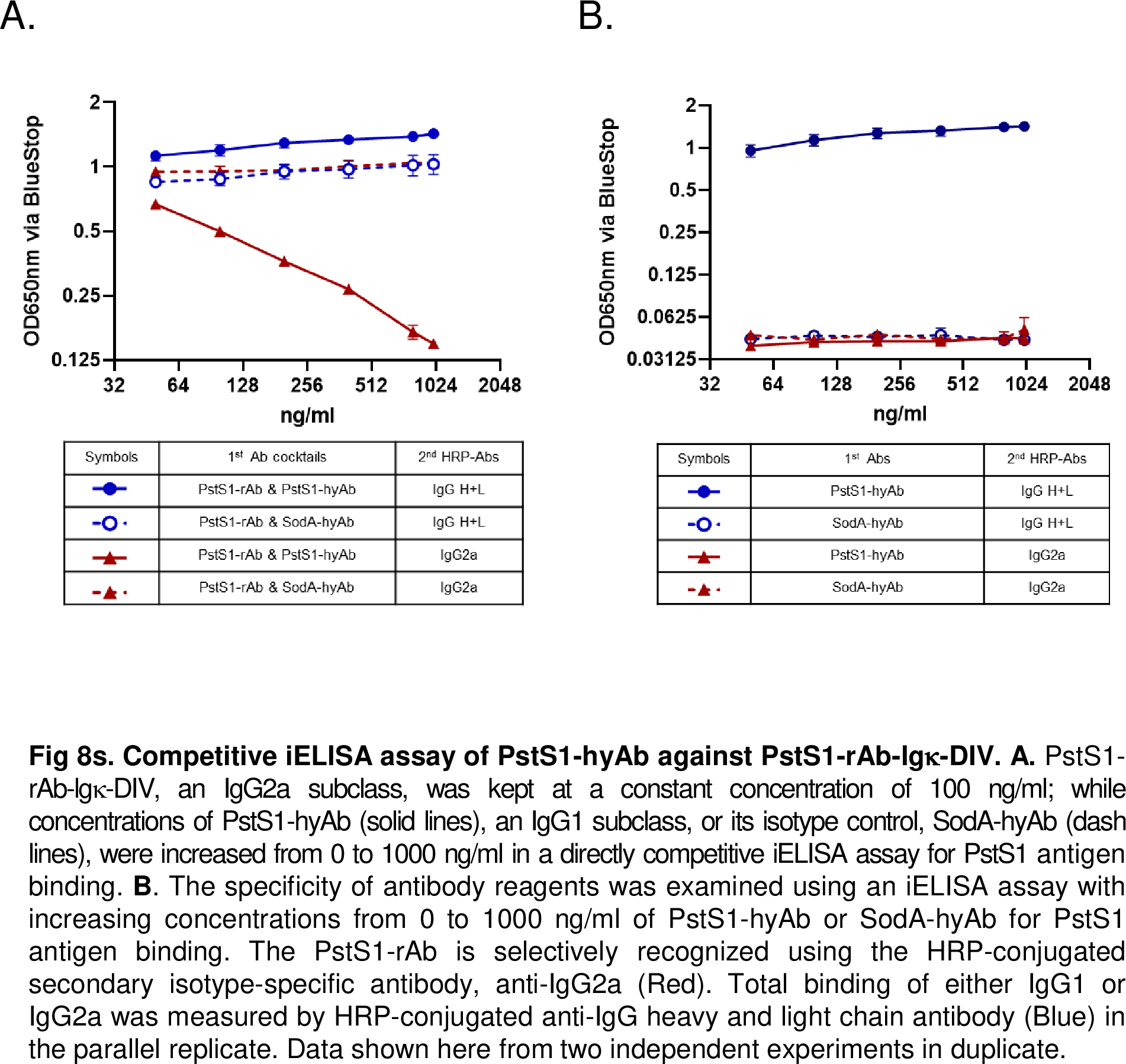

Supplement: S8 Fig — A. PstS1-rAb-lgk-DIV, an IgG2a subclass, was kept at a constant concentration of 100 ng/ml; while concentrations of PstS1-hyAb (solid lines), an IgG1 subclass, or its isotype control, SodA-hyAb (dash lines), were increased from 0 to 1000 ng/ml in a directly competitive iELISA assay for PstS1 antigen binding. B. The specificity of antibody reagents was examined using an iELISA assay with increasing concentrations from 0 to 1000 ng/ml of PstS1-hyAb or SodA-hyAb for PstS1 antigen binding. The PstS1-rAb is selectively recognized using the HRP-conjugated secondary isotype-specific antibody, anti-IgG2a (Red). Total binding of either IgG1 or IgG2a was measured by HRP-conjugated anti-IgG heavy and light chain antibody (Blue) in the parallel replicate. Data shown here from two independent experiments in duplicate. (TIF) [file pone.0256079.s008.tif]

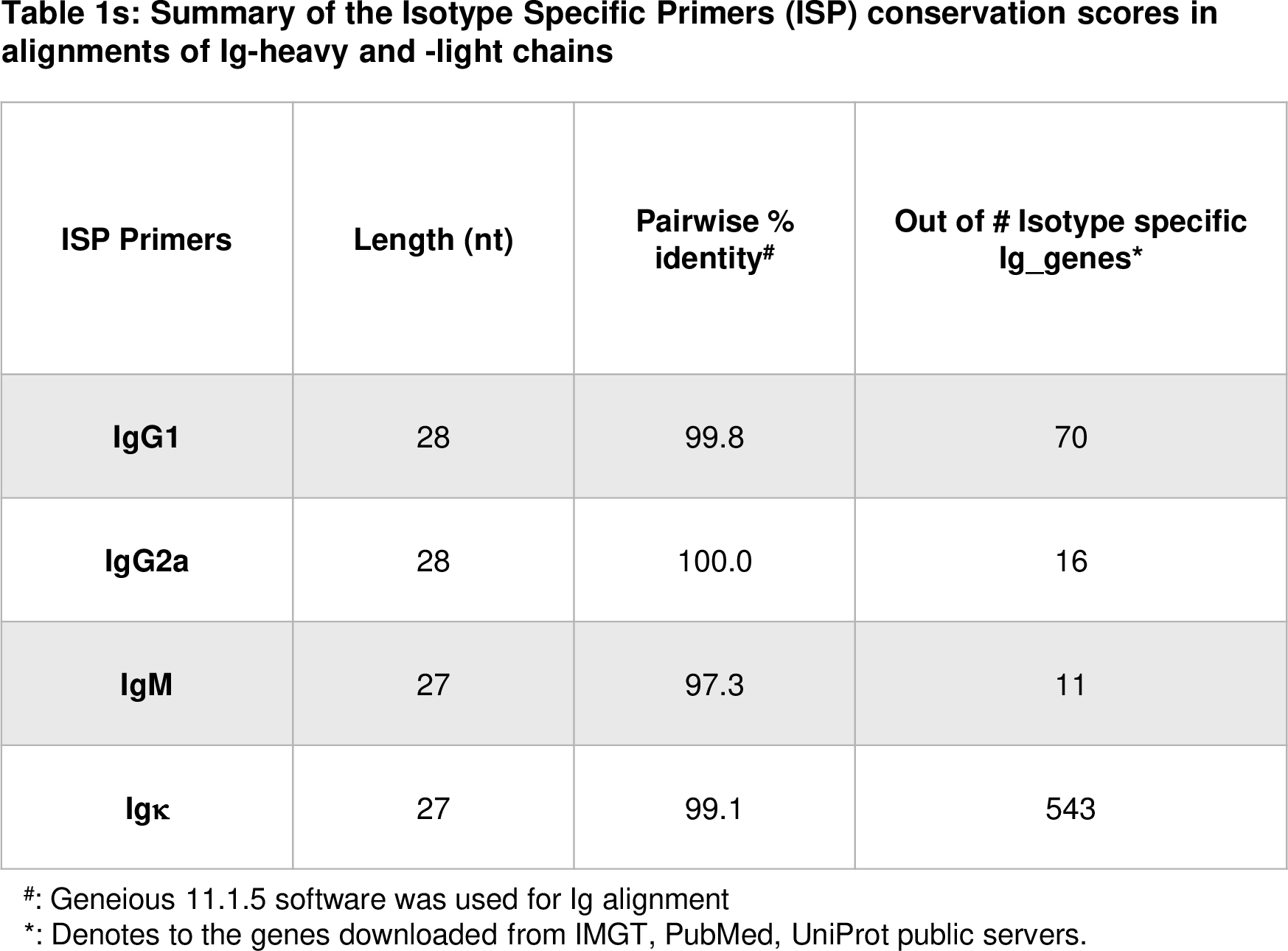

Supplement: S1 Table — (TIF) [file pone.0256079.s009.tif]

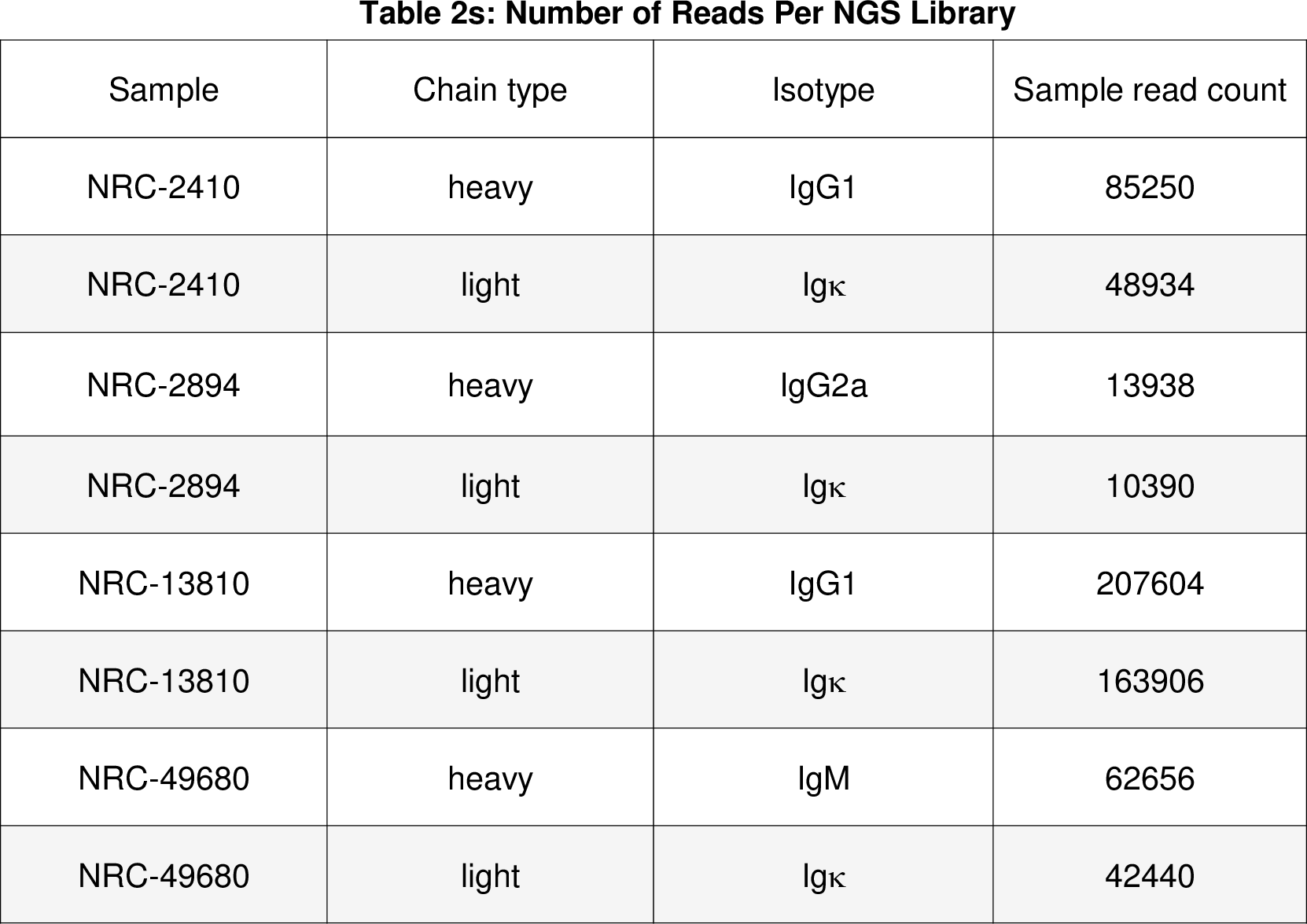

Supplement: S2 Table — (TIF) [file pone.0256079.s010.tif]

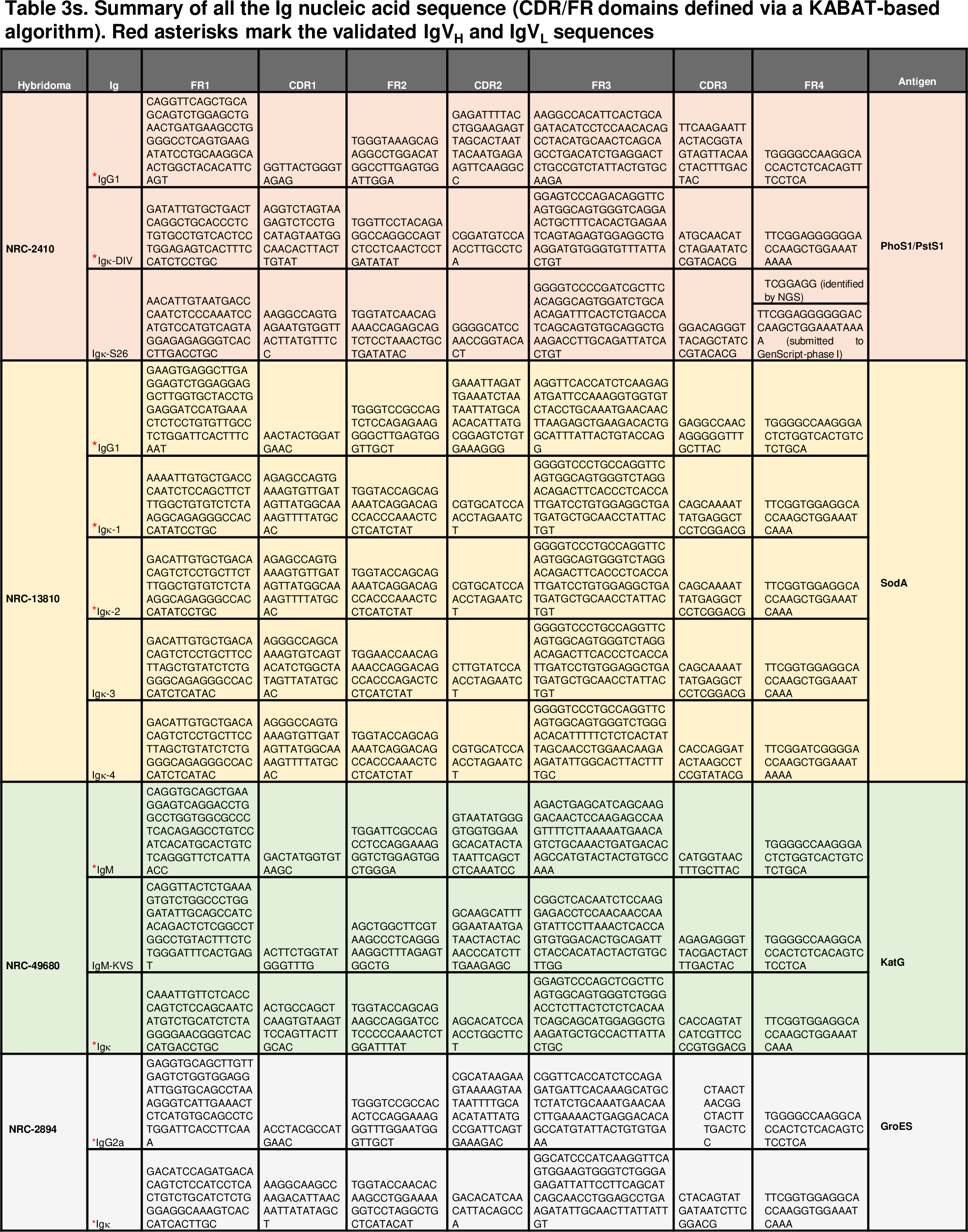

Supplement: S3 Table — Red asterisks mark the validated IgVH and IgVL sequences. (TIF) [file pone.0256079.s011.tif]

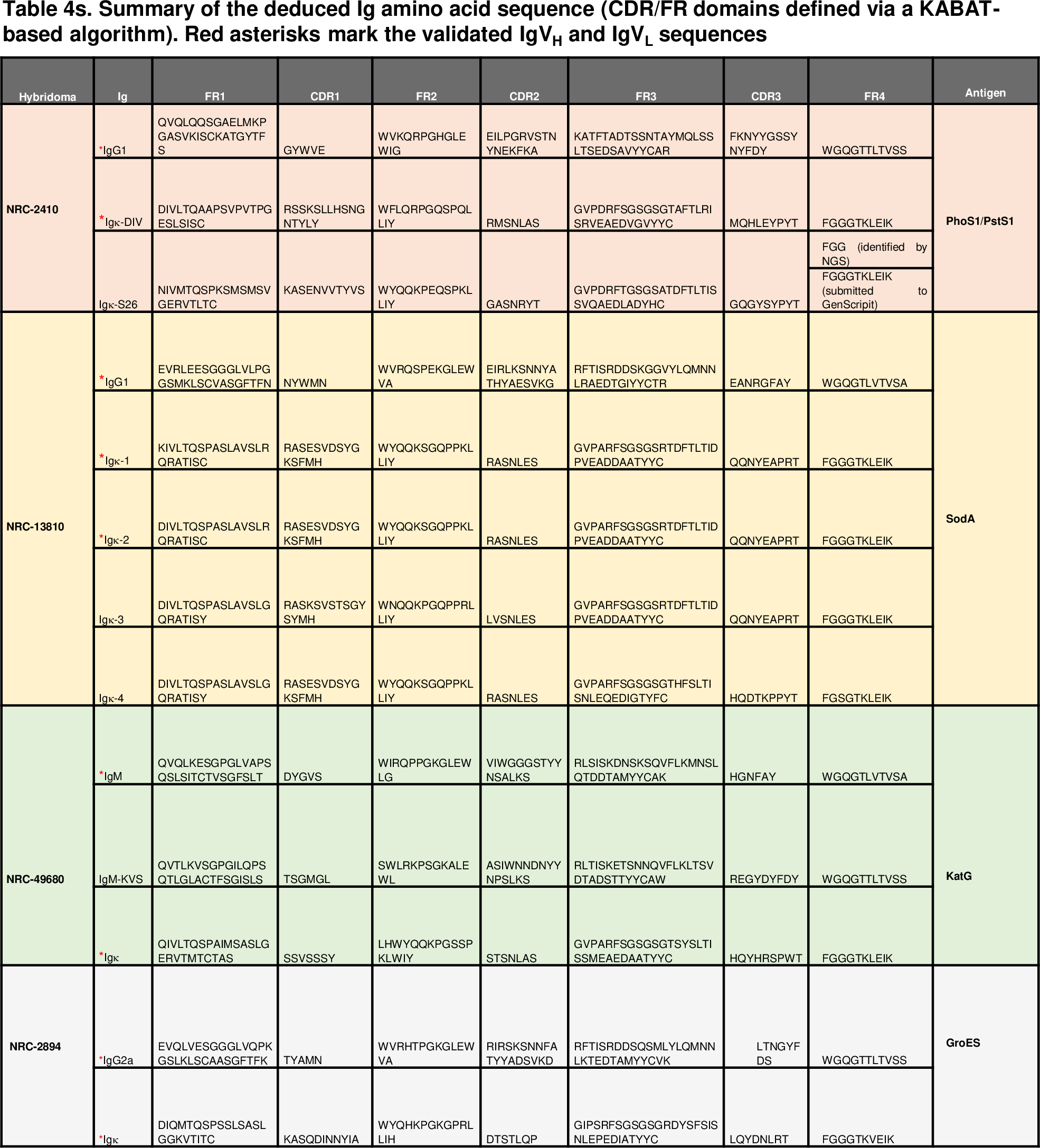

Supplement: S4 Table — Red asterisks mark the validated IgVH and IgVL sequences. (TIF) [file pone.0256079.s012.tif]

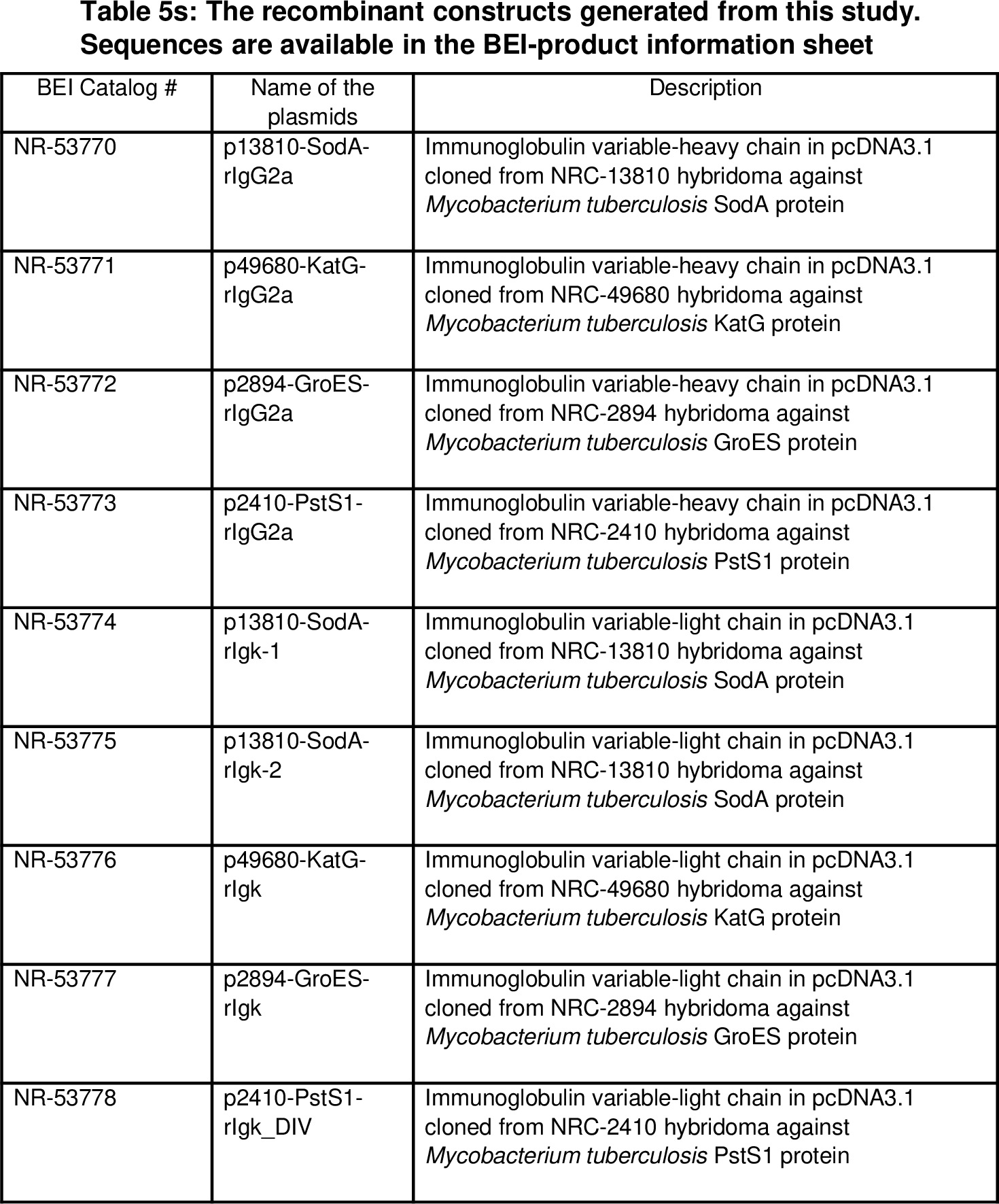

Supplement: S5 Table — Sequences are available in the BEI-product information sheet. (TIF) [file pone.0256079.s013.tif]

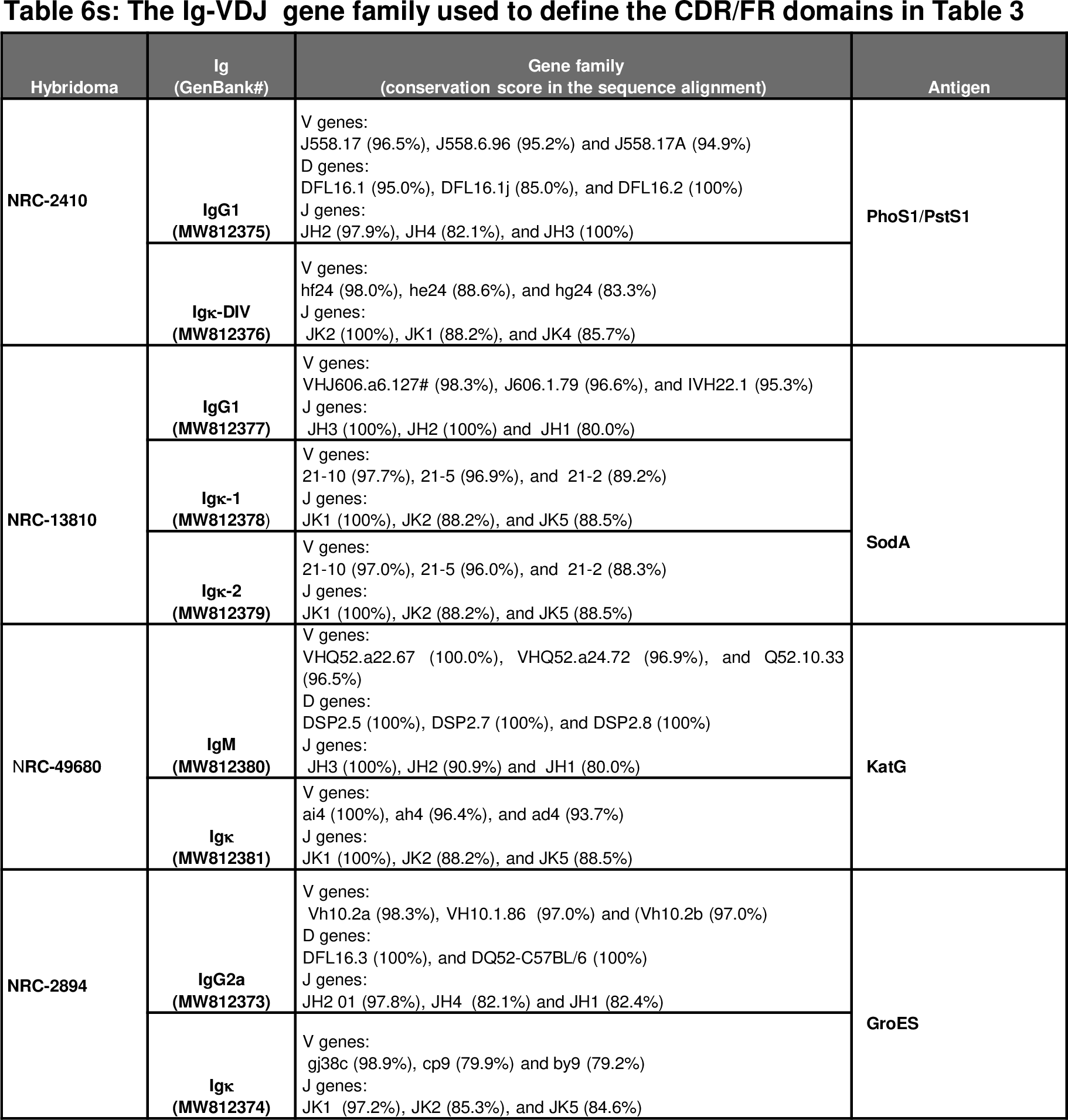

Supplement: S6 Table — (TIF) [file pone.0256079.s014.tif]

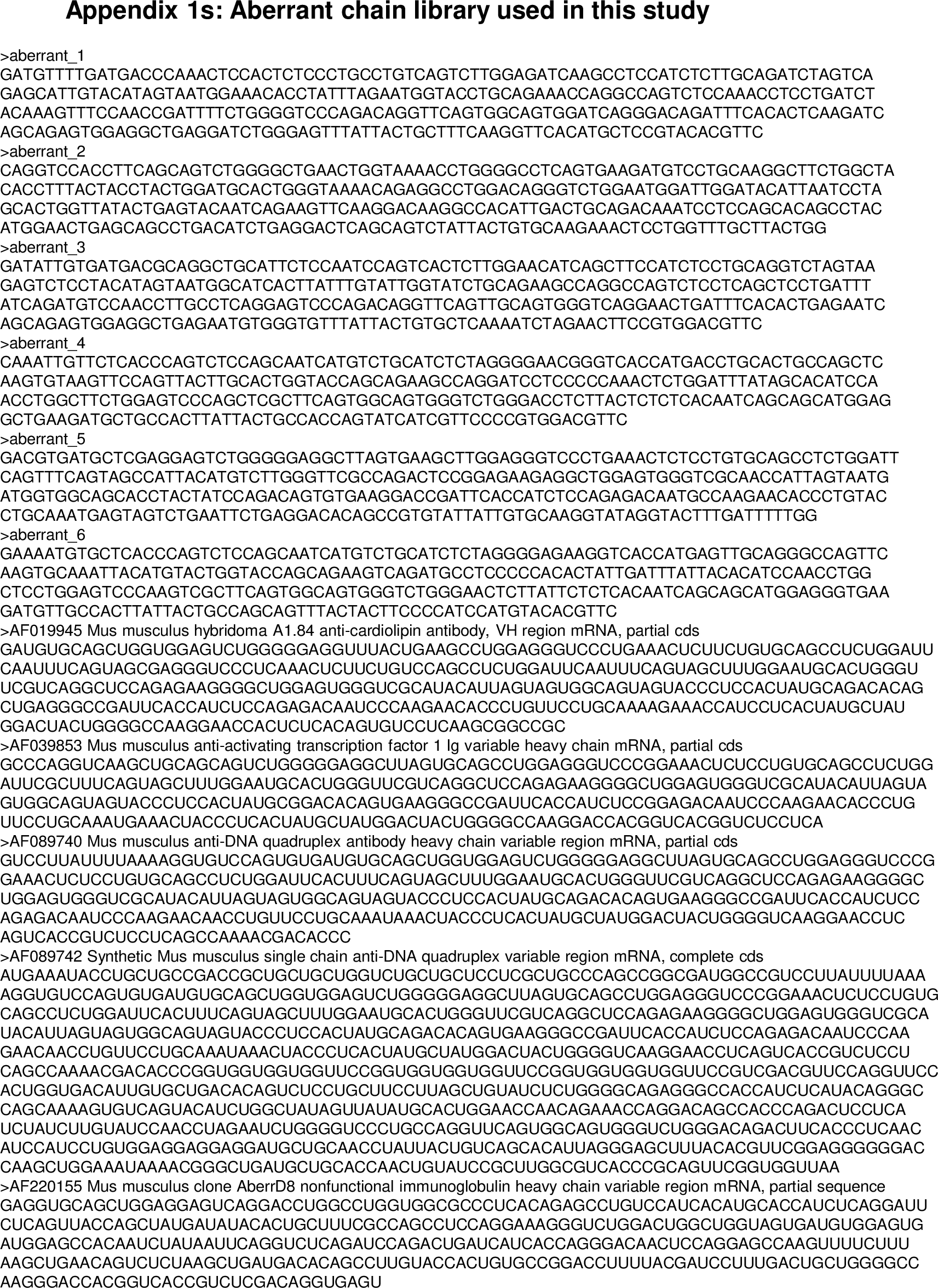

Supplement: S1 Appendix — (ZIP) [file pone.0256079.s015.zip › S1_Appendix.tif]

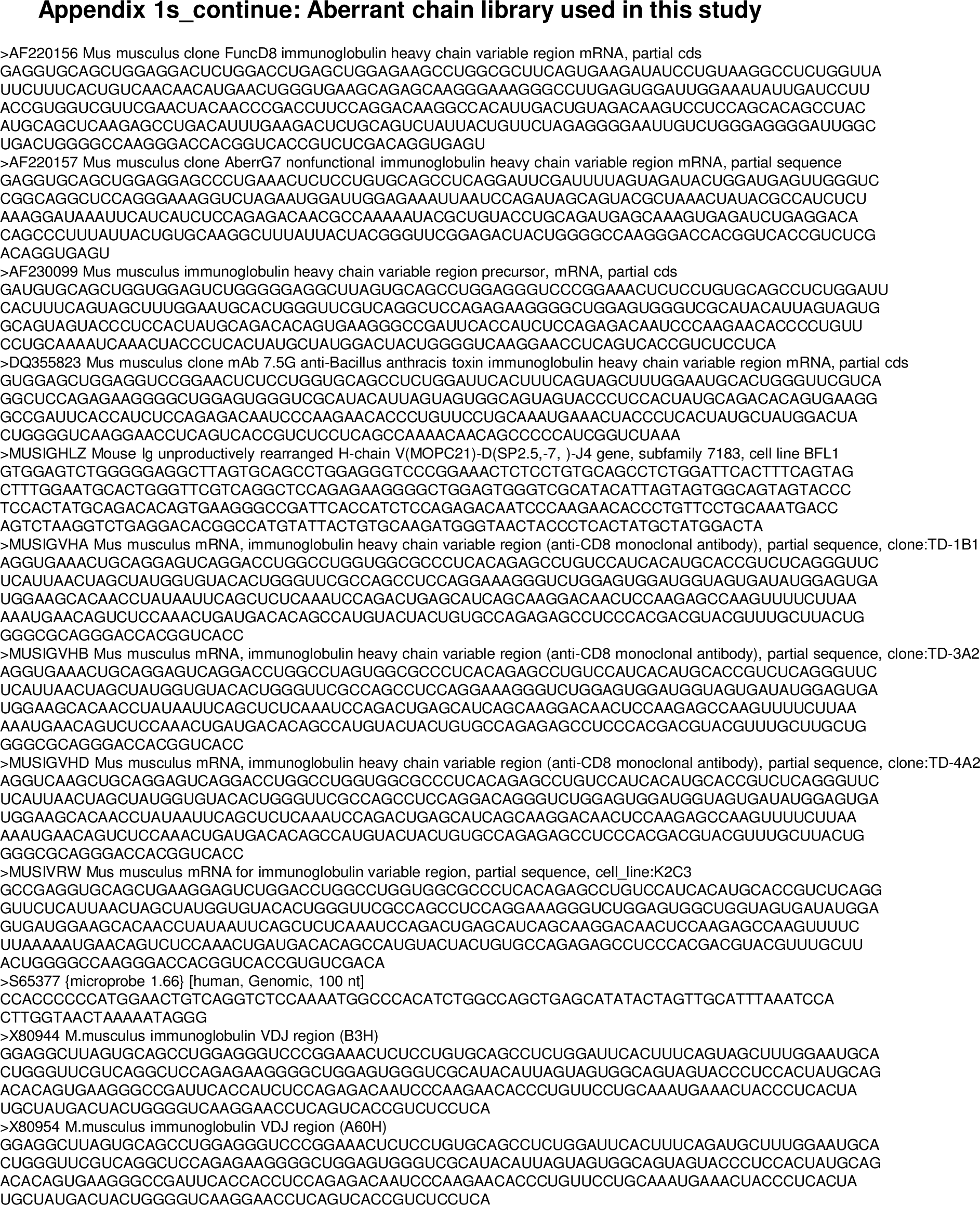

Supplement: S1 Appendix — (ZIP) [file pone.0256079.s015.zip › S1_Appendix_continue.tif]

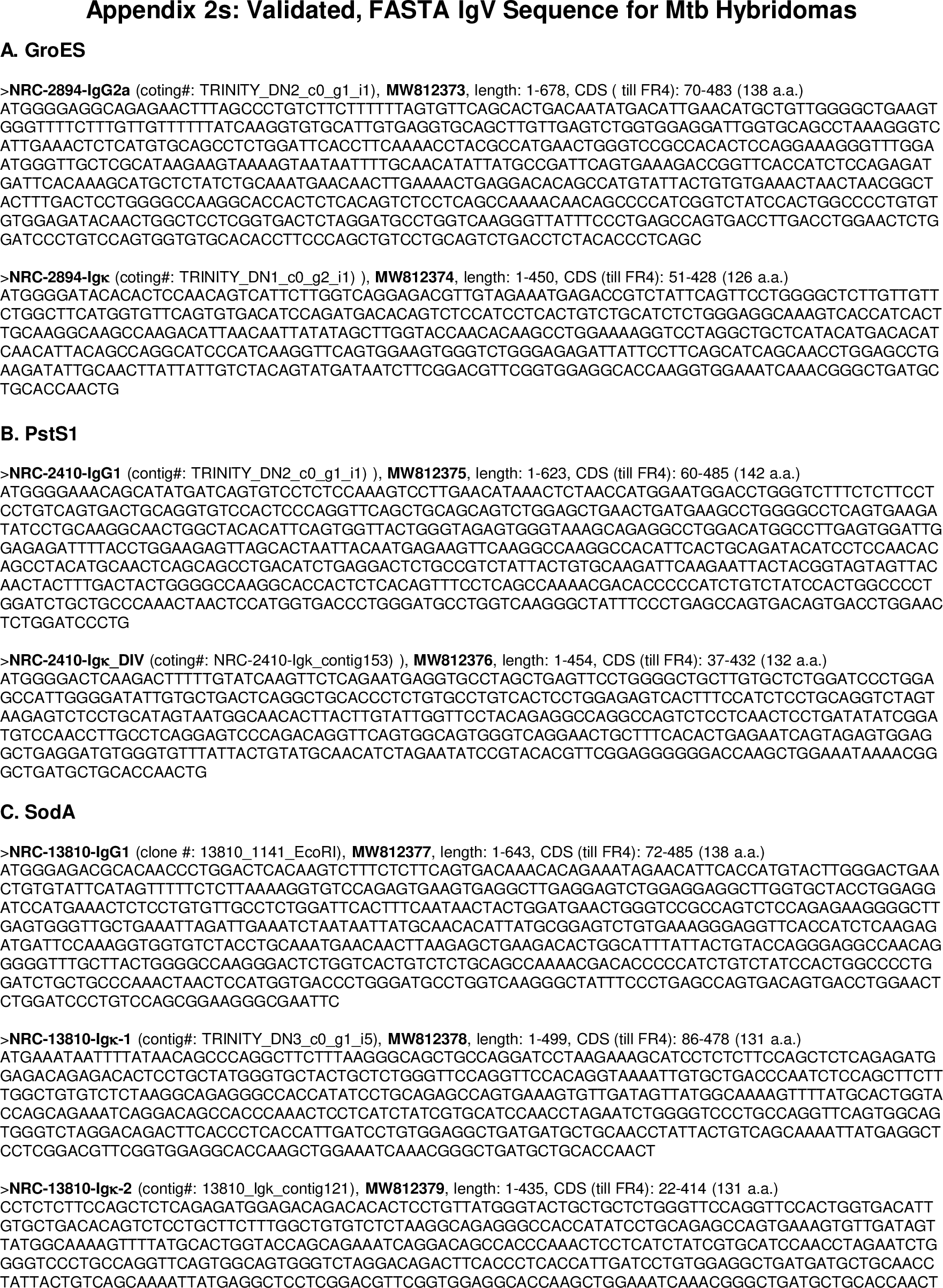

Supplement: S2 Appendix — (ZIP) [file pone.0256079.s016.zip › S2_Appendix.tif]

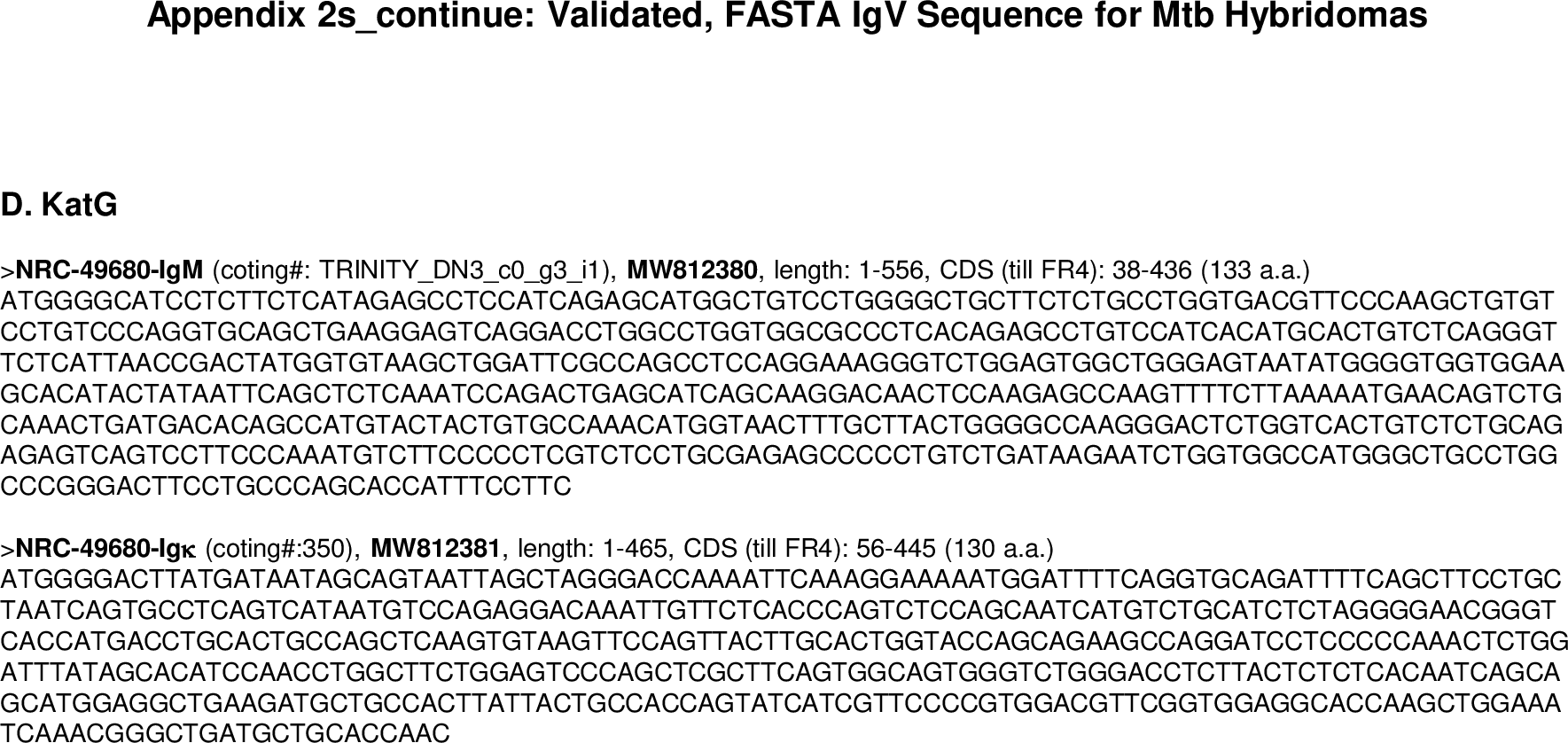

Supplement: S2 Appendix — (ZIP) [file pone.0256079.s016.zip › S2_Appendix_continue.tif]

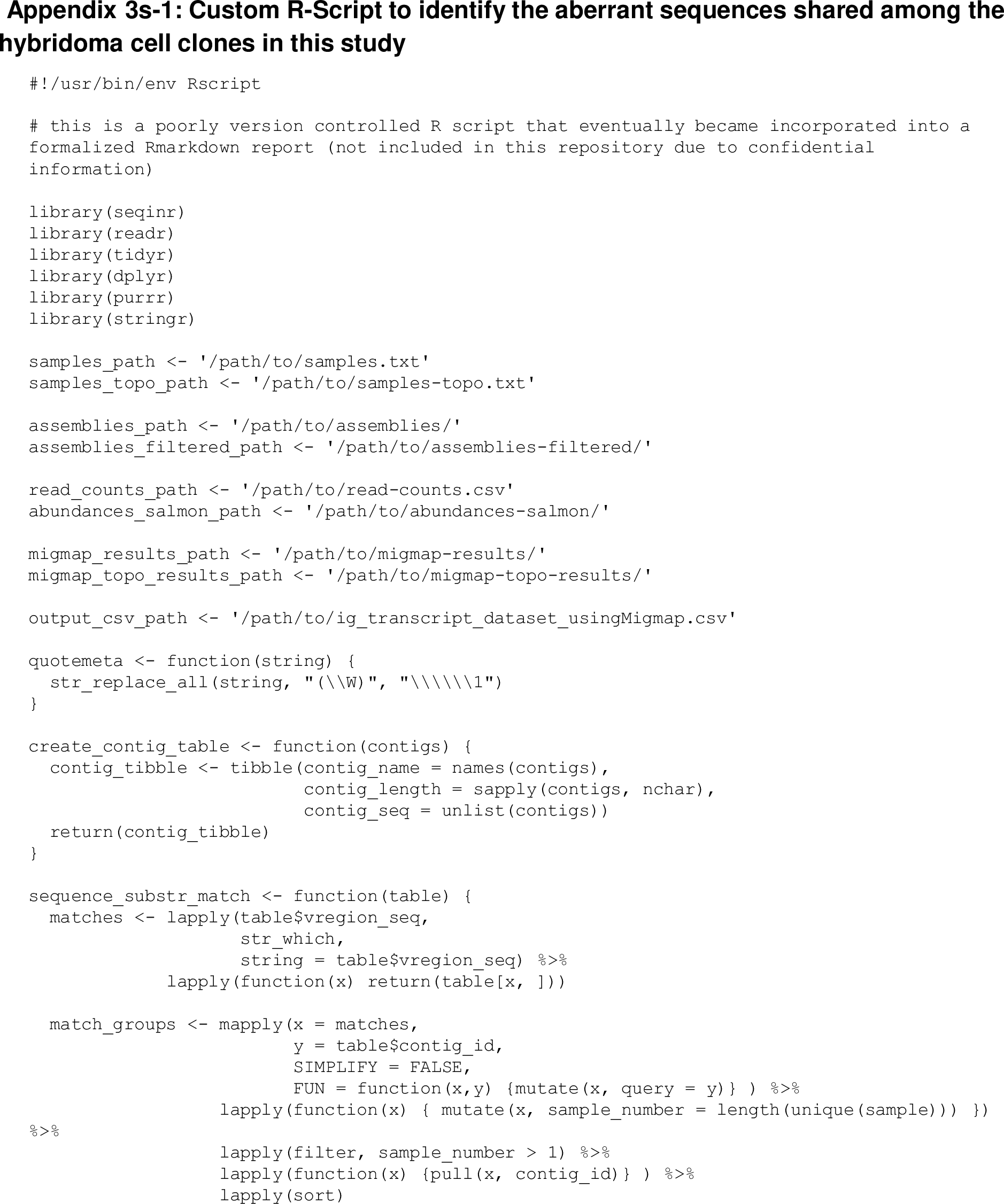

Supplement: S3 Appendix — (ZIP) [file pone.0256079.s017.zip › S3-1_Appendix.tif]

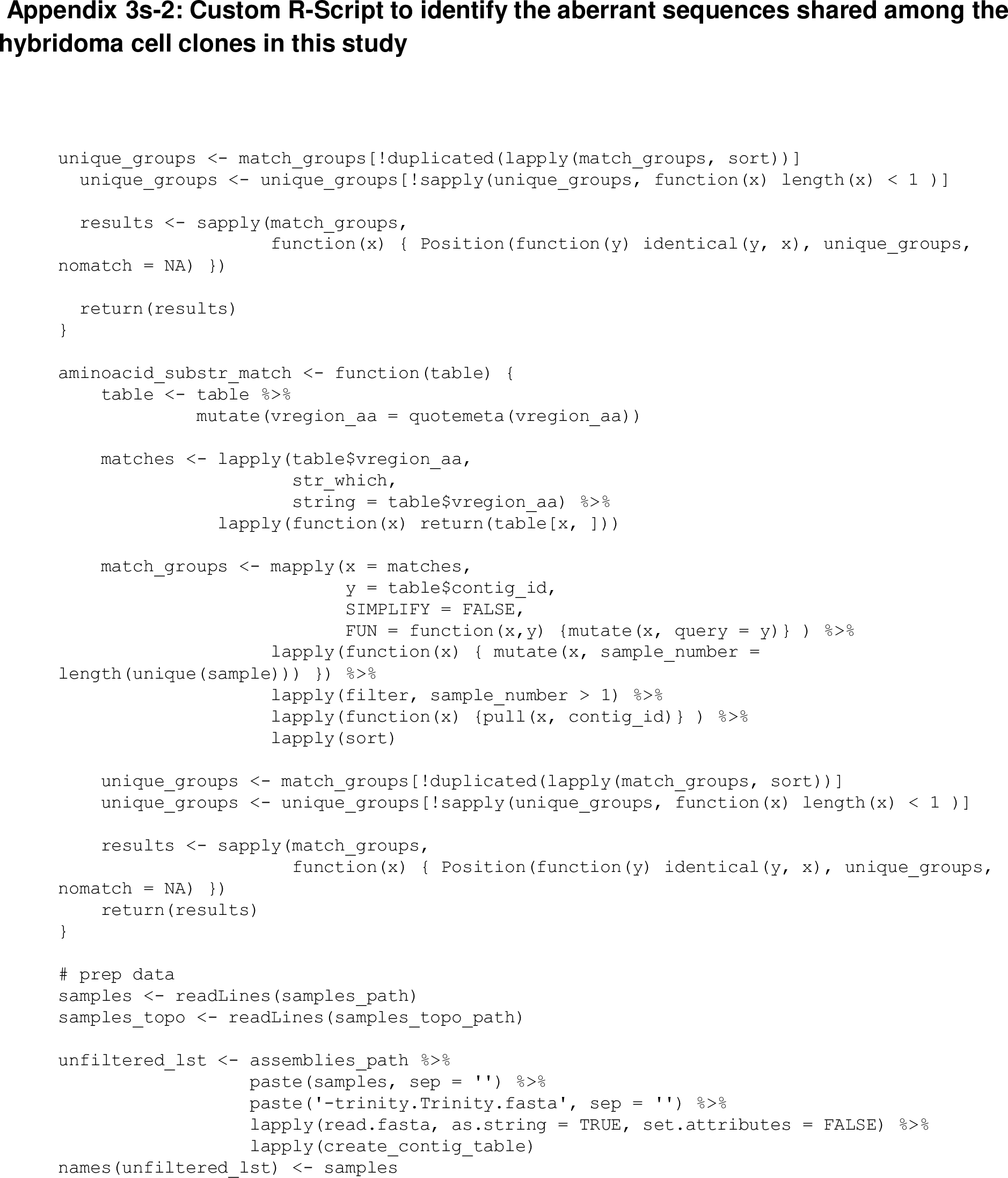

Supplement: S3 Appendix — (ZIP) [file pone.0256079.s017.zip › S3-2_Appendix.tif]

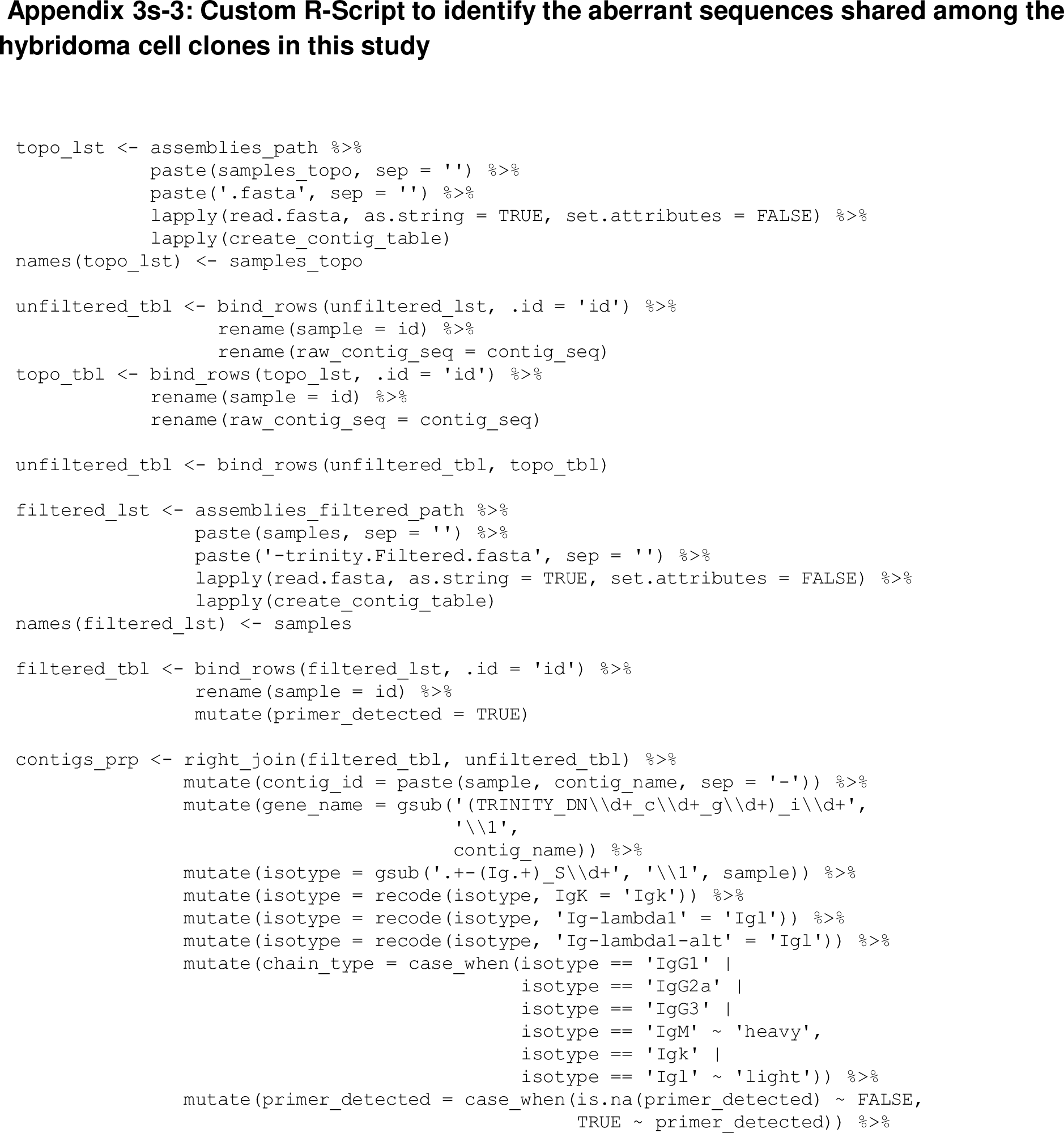

Supplement: S3 Appendix — (ZIP) [file pone.0256079.s017.zip › S3-3_Appendix.tif]

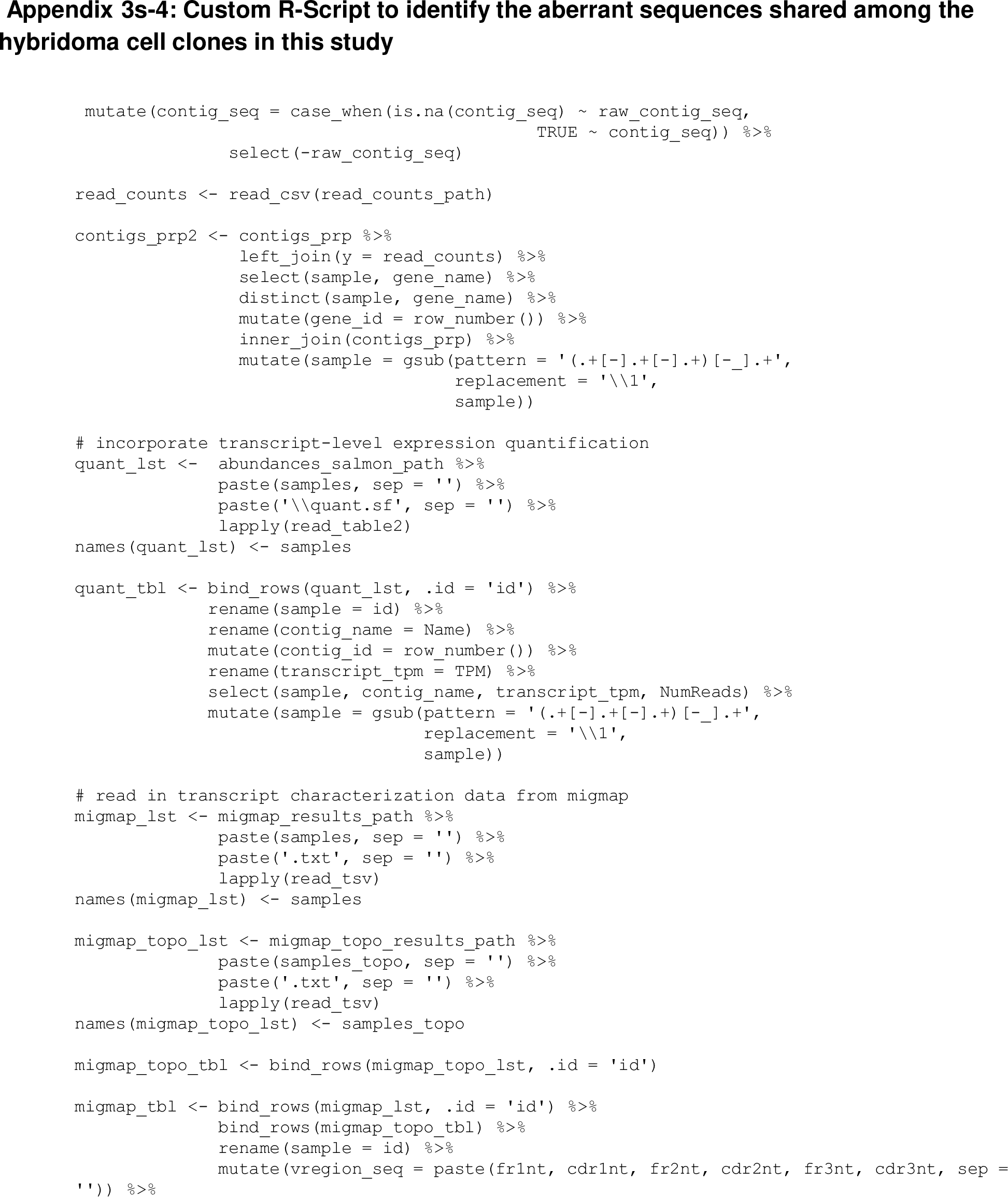

Supplement: S3 Appendix — (ZIP) [file pone.0256079.s017.zip › S3-4_Appendix.tif]

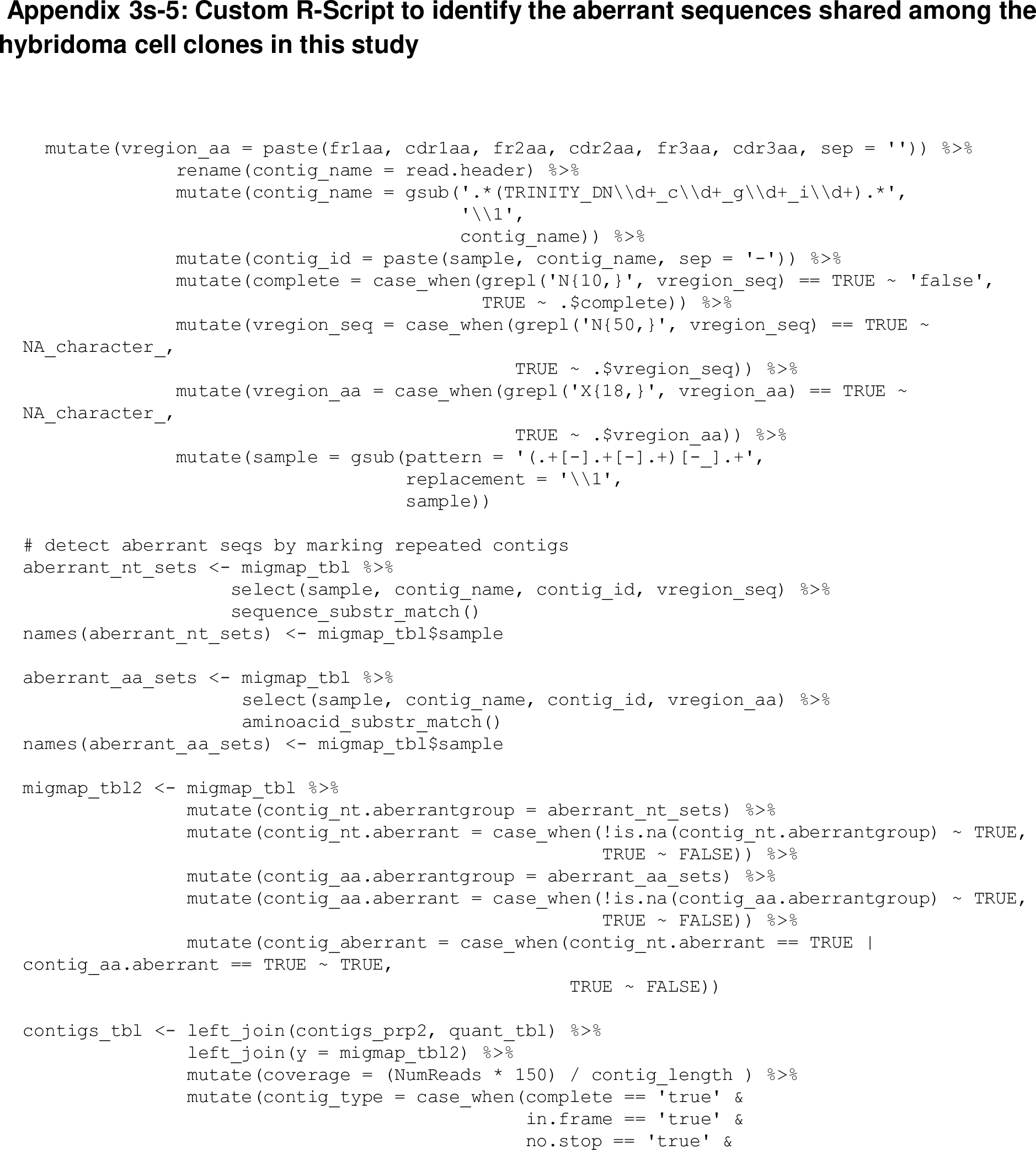

Supplement: S3 Appendix — (ZIP) [file pone.0256079.s017.zip › S3-5_Appendix.tif]

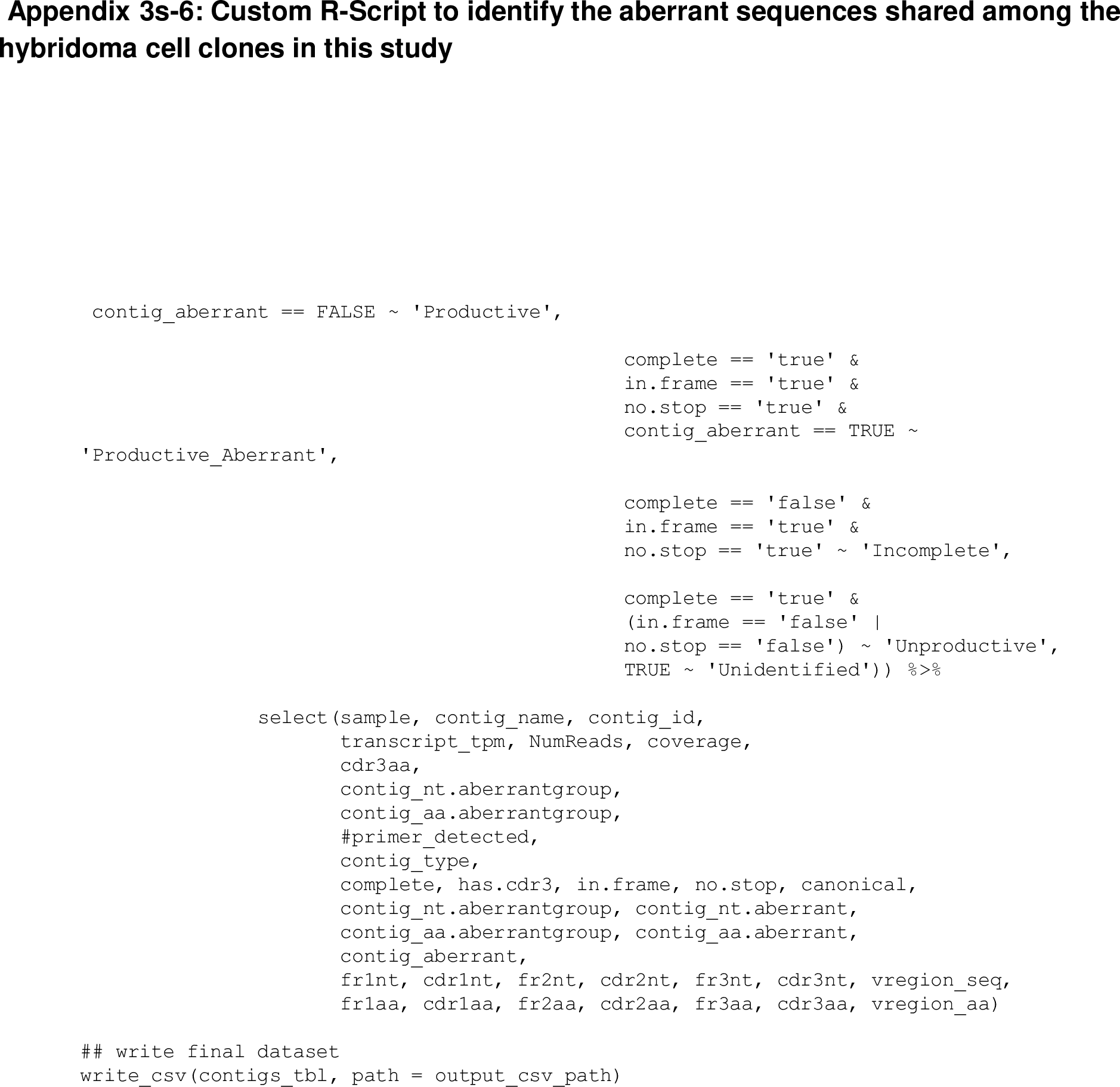

Supplement: S3 Appendix — (ZIP) [file pone.0256079.s017.zip › S3-6_Appendix.tif]
