# Supplementary material for: Determination of variable region sequences from hybridoma immunoglobulins that target Mycobacterium tuberculosis virulence factors
Source: PLoS One. 2021 Aug 20;16(8):e0256079. doi: 10.1371/journal.pone.0256079 (PMC8378720; doi:10.1371/journal.pone.0256079)
Supplement: S1 Raw images — (PDF) [file pone.0256079.s018.pdf]

Figures with Original blots

Determination of Variable Region Sequences from Hybridoma Immunoglobulins that Target Mycobacterium tuberculosis Virulence Factors

Hui-Chen Chang Foreman, Andrew Frank, and Timothy T. Stedman

BEI Resources, ATCC. 10801 University Boulevard, Manassas, VA 20110-2209

Final Fig 1B

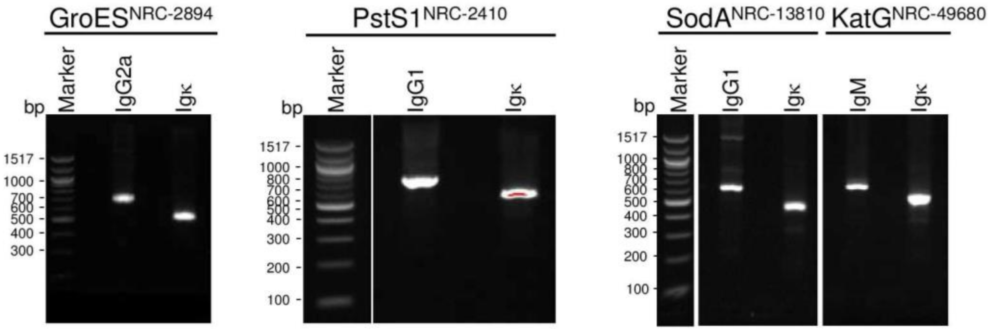

Original gel/blot pictures

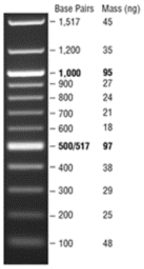

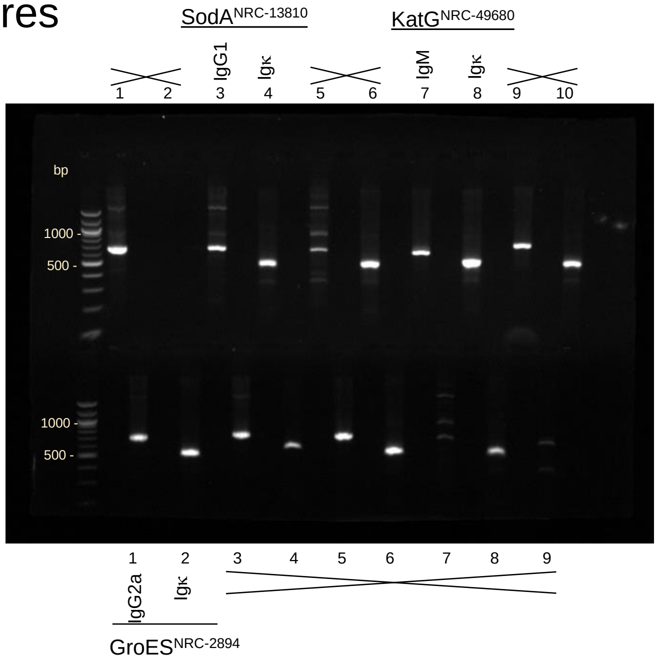

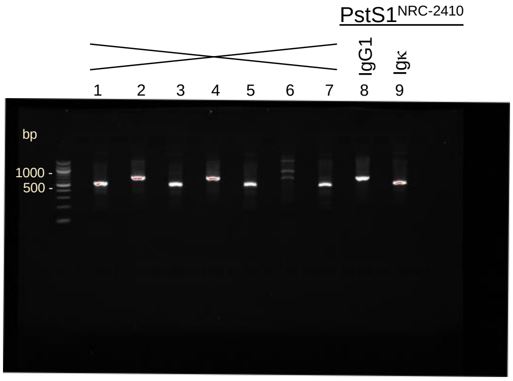

Final Fig 3B

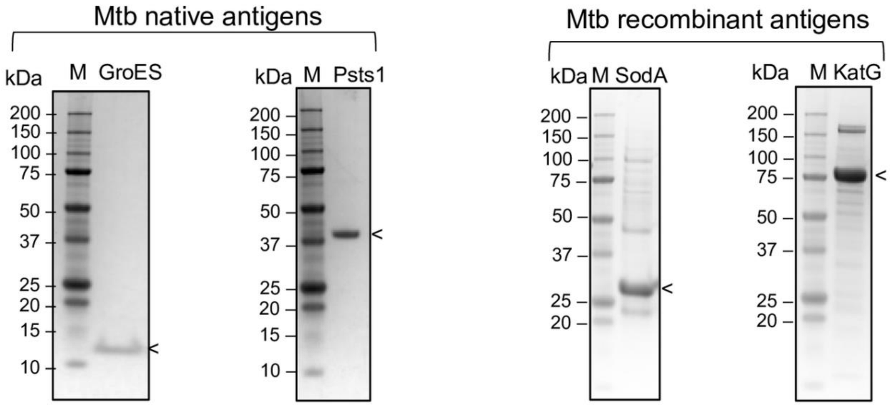

Original gel/blot pictures

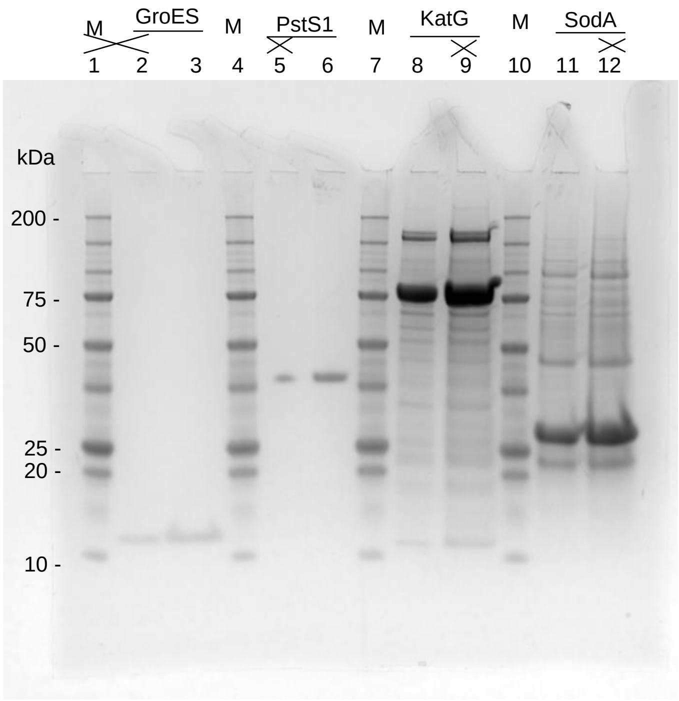

Final Fig 3C & D

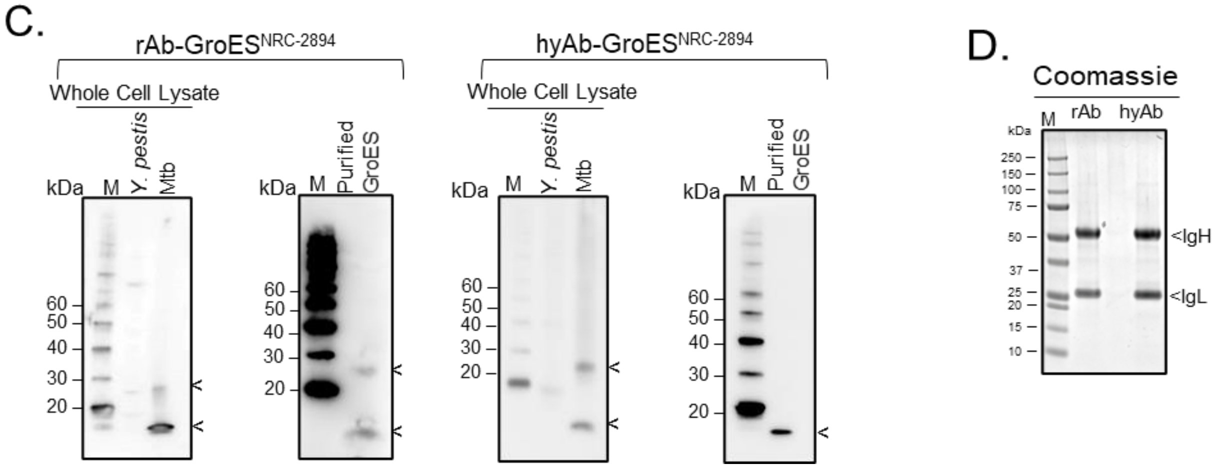

Original gel/blot pictures

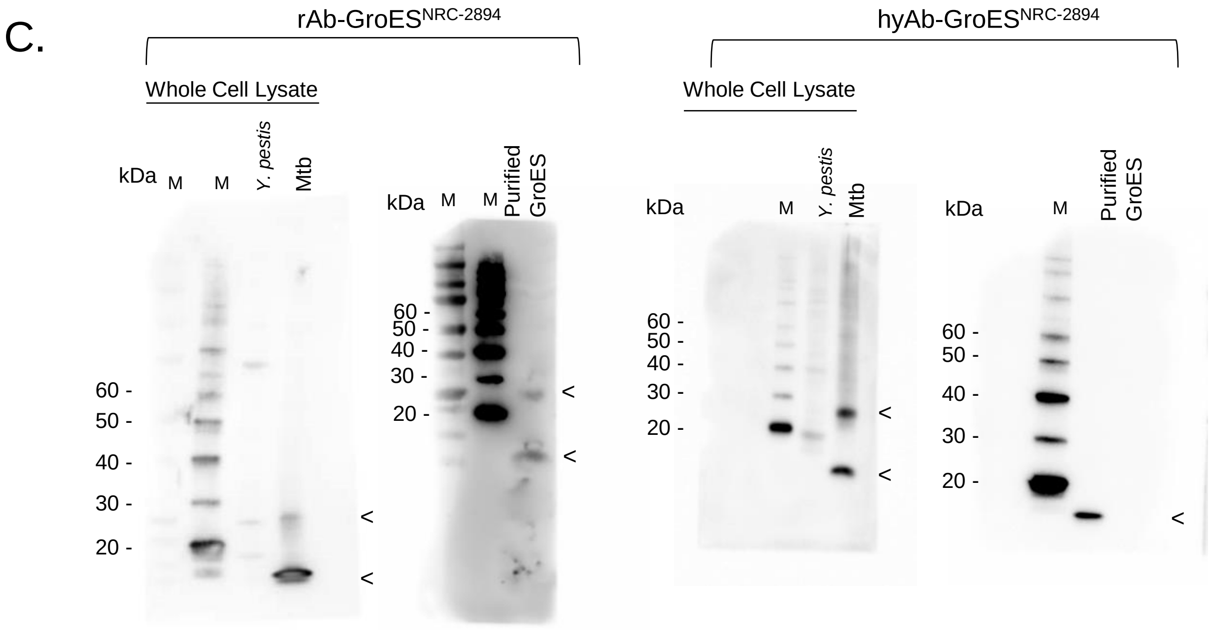

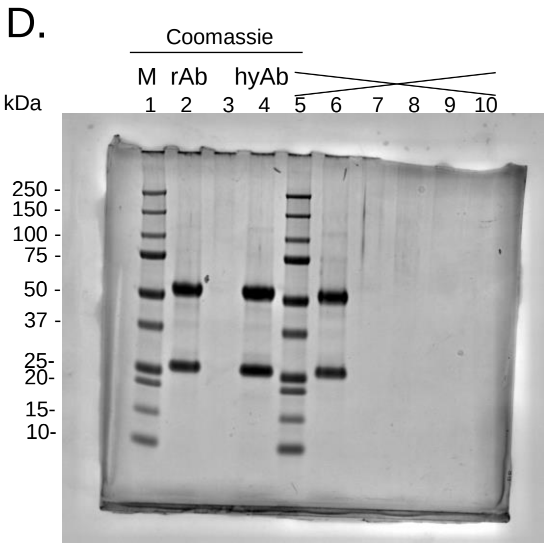

Final Fig 4A

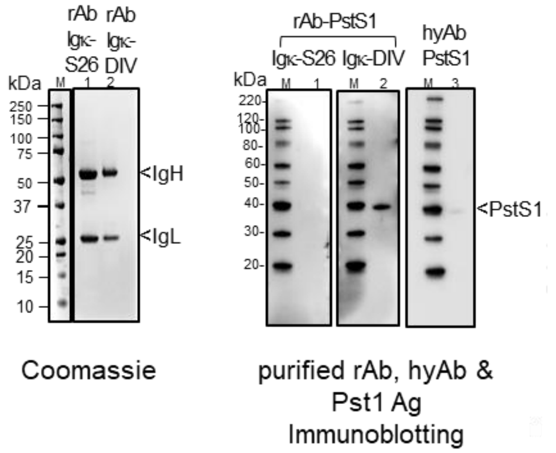

Original gel/blot pictures

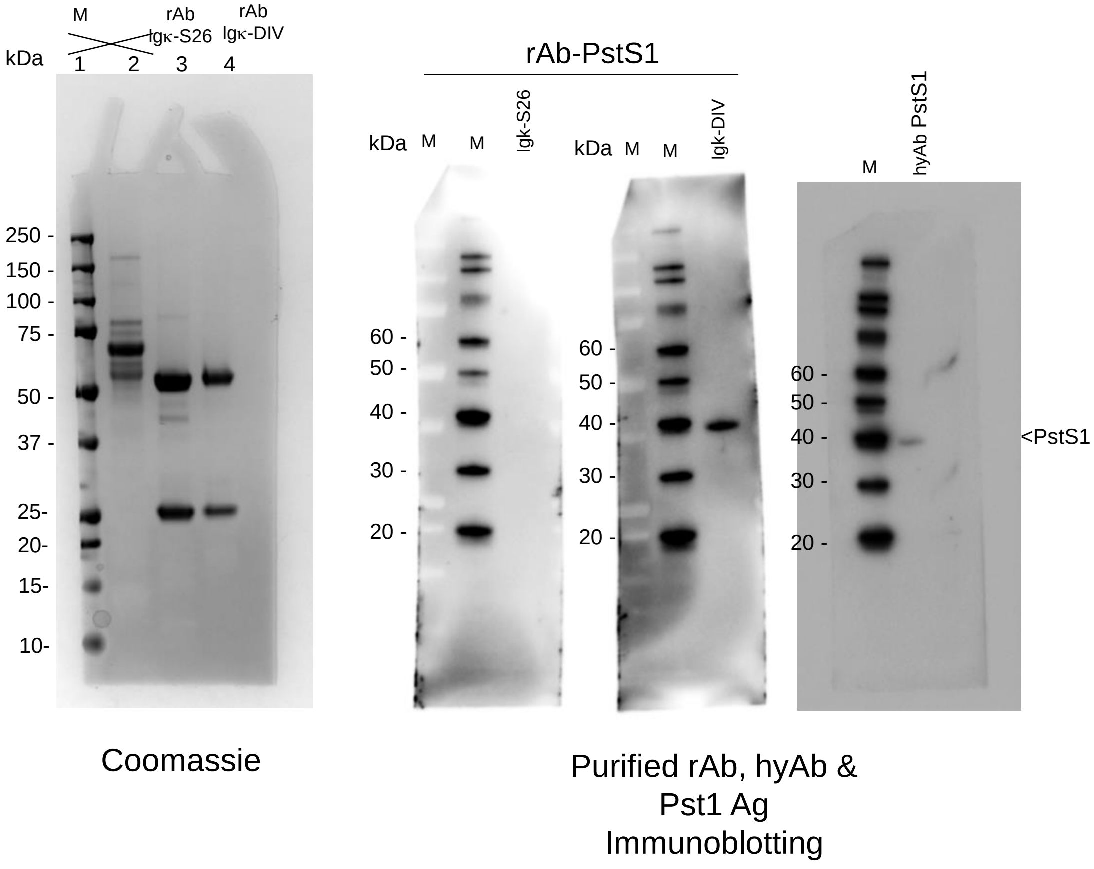

Final Fig 4B

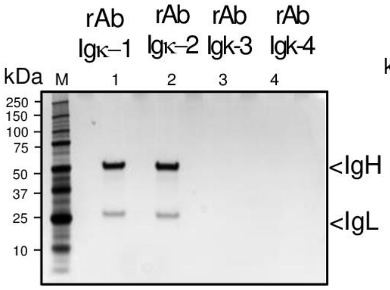

Silver stain

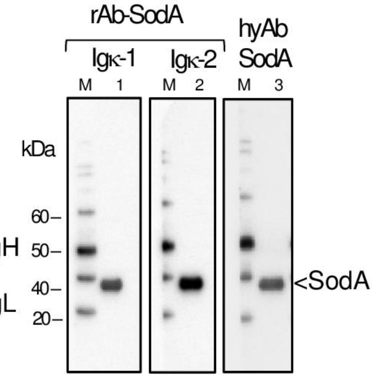

purified rAb, hyAb & SodA Ag Immunoblotting

Original gel/blot pictures

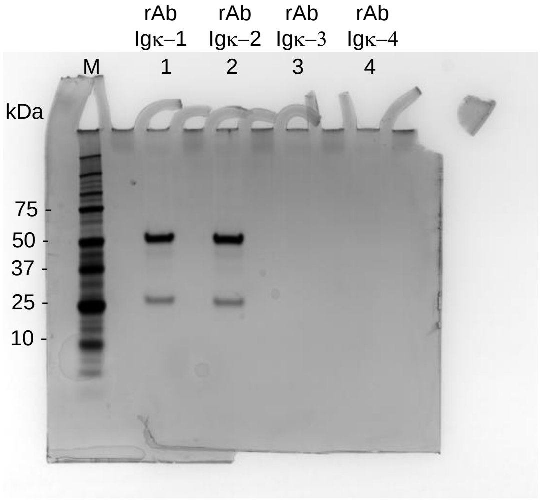

Silver stain

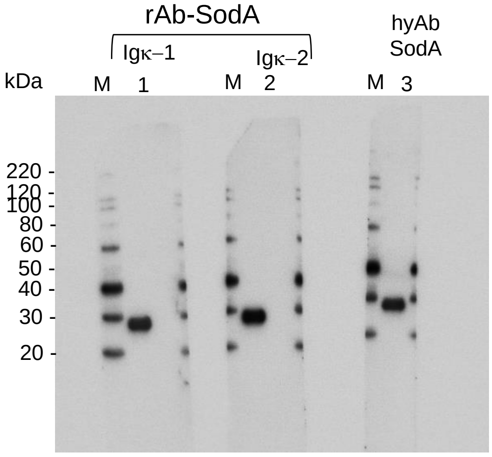

Immunoblotting

Final Fig 4C

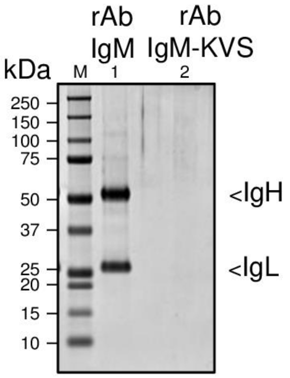

Coomassie

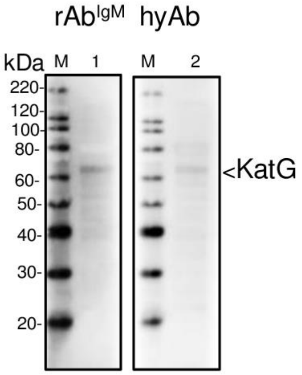

purified rAb, hyAb &
KatG Ag
Immunoblotting

Original gel/blot pictures

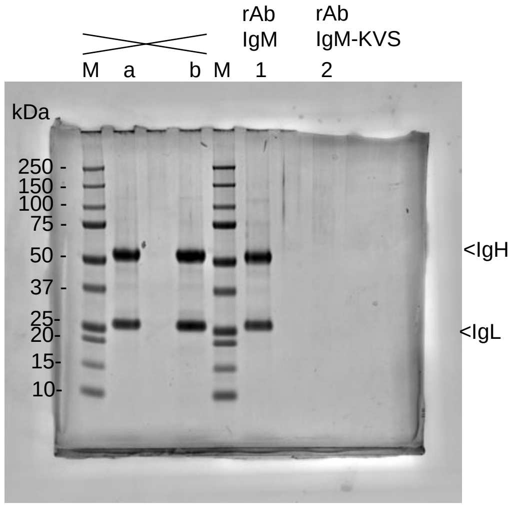

Silver stain

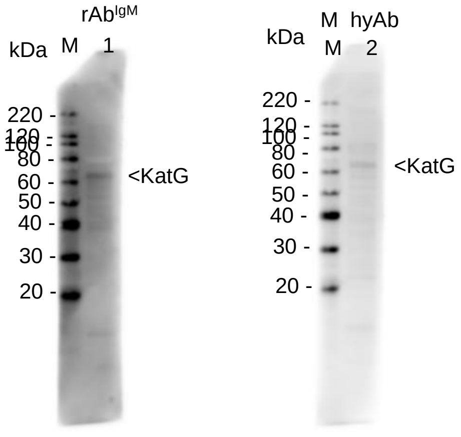

Immunoblotting

Supplemental

Final Fig 1s-C

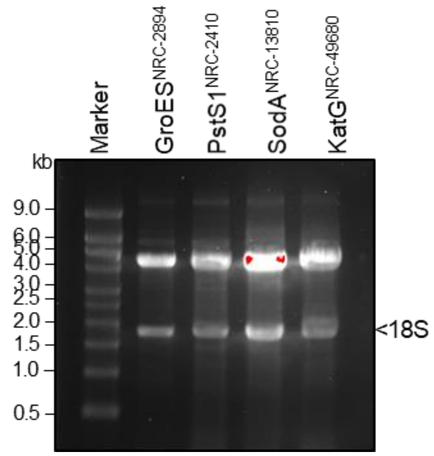

Original gel/blot pictures

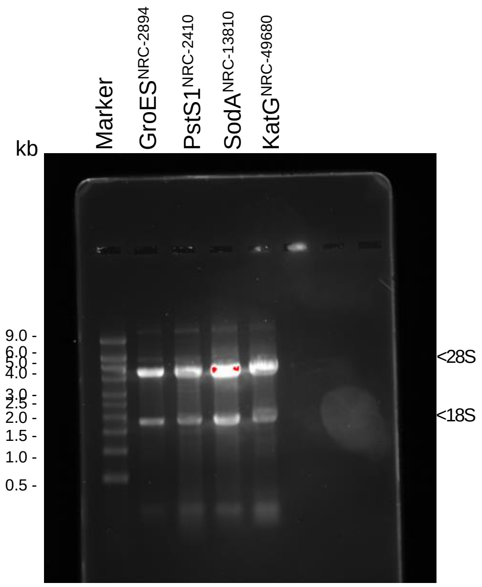

Final Fig 1s-D

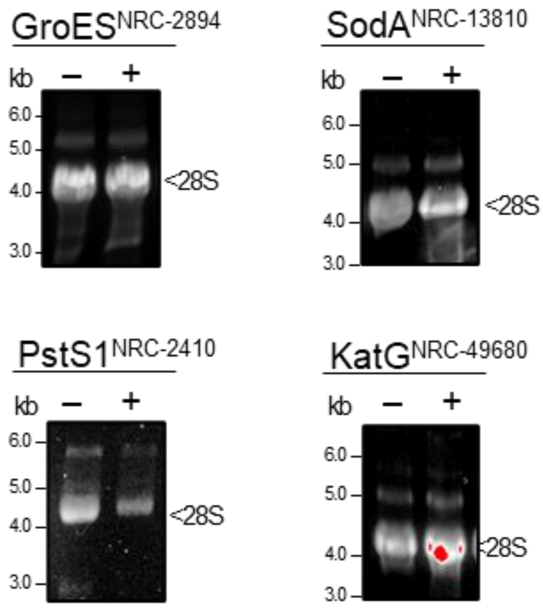

Original gel/blot pictures

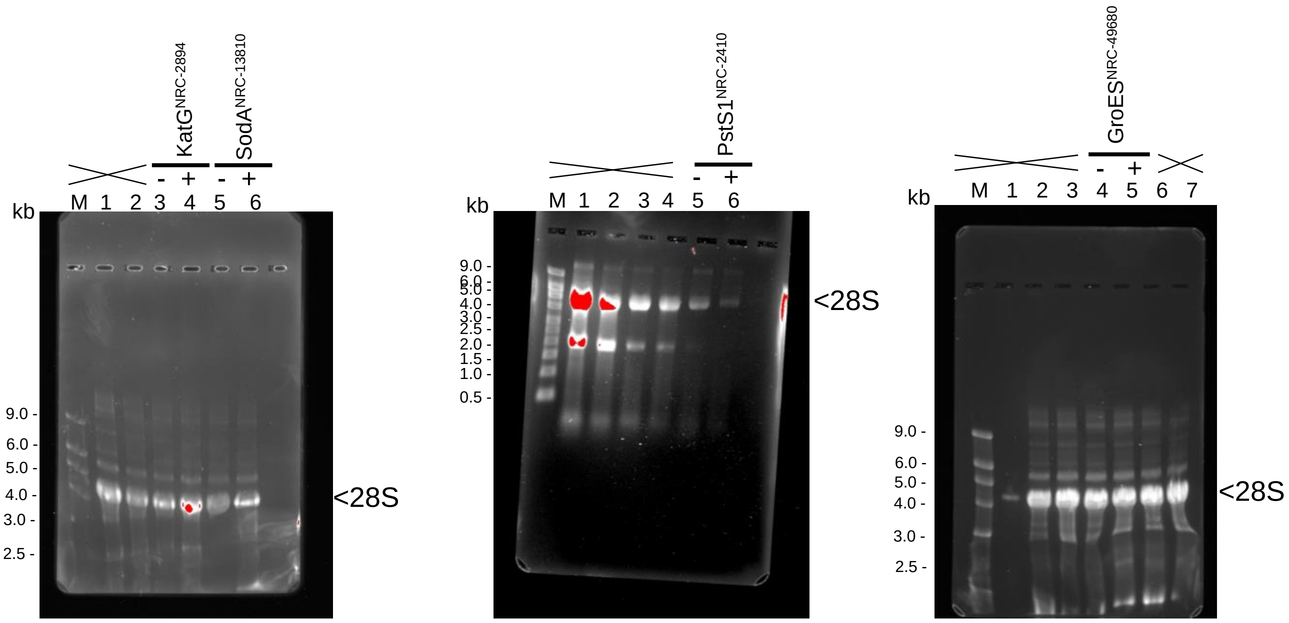

Final Fig 3s

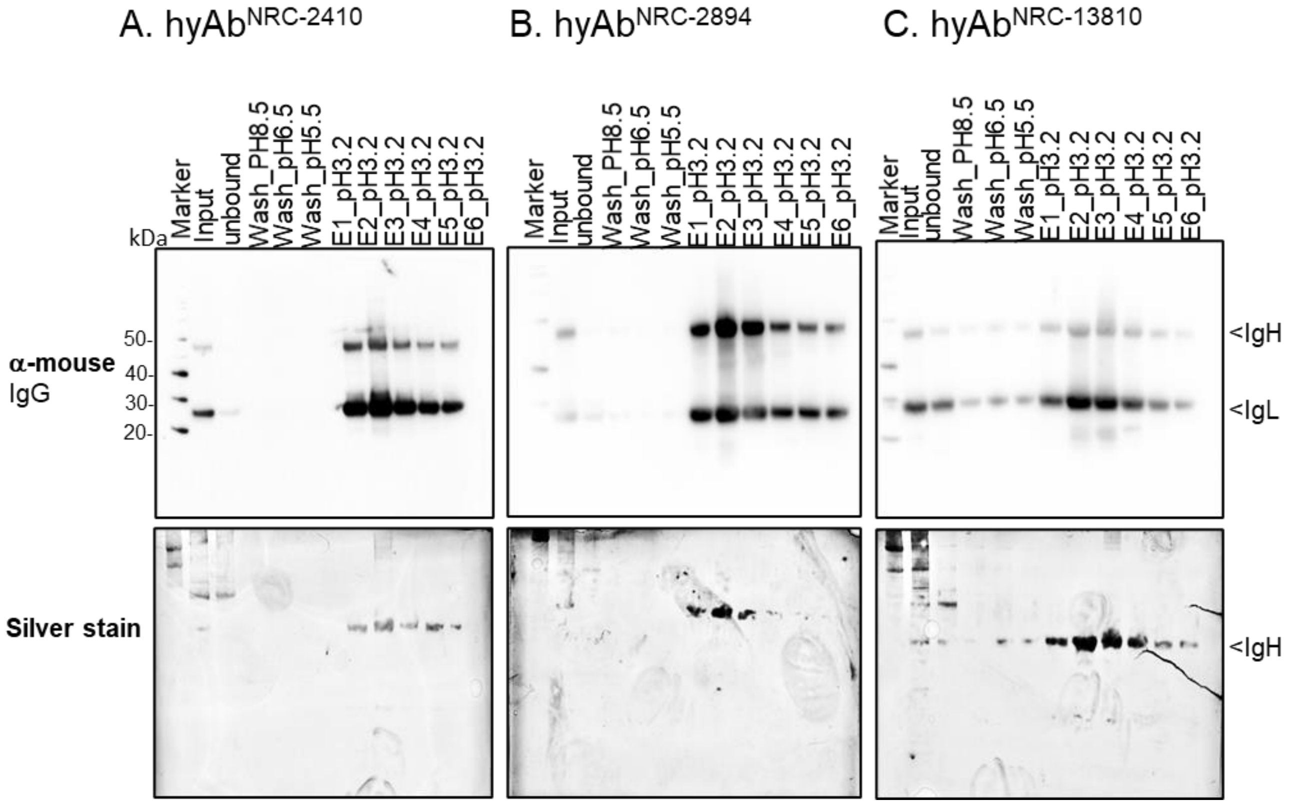

Original gel/blot pictures

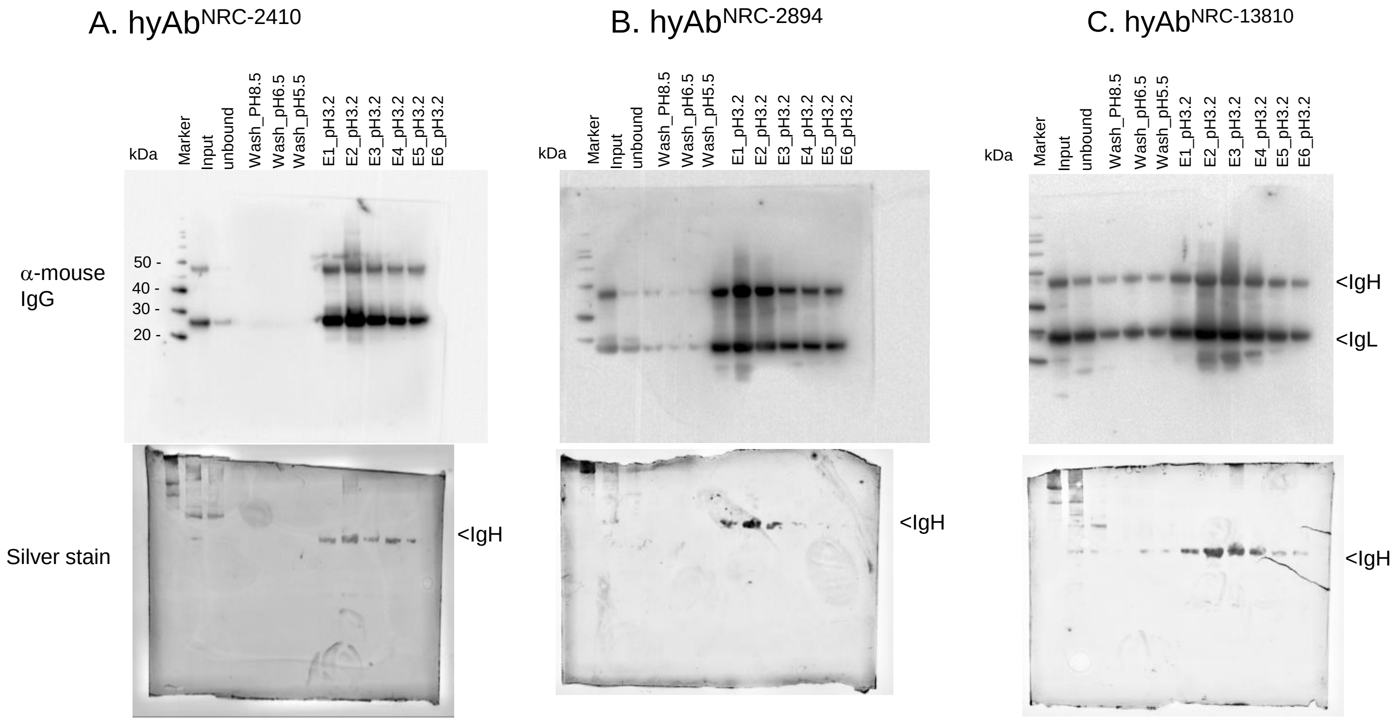

Top α–mouse IgG panel lists the blots developed by ECL and captured by Bio-Rad Image Lab 6.0 with adjustable brightness transformation. The image of the original blots is shown with higher saturated pixels than the final figures for an easy view of the gel boundary. Note that every image is from a whole, un-crop gel.

Final Figure 4s-A

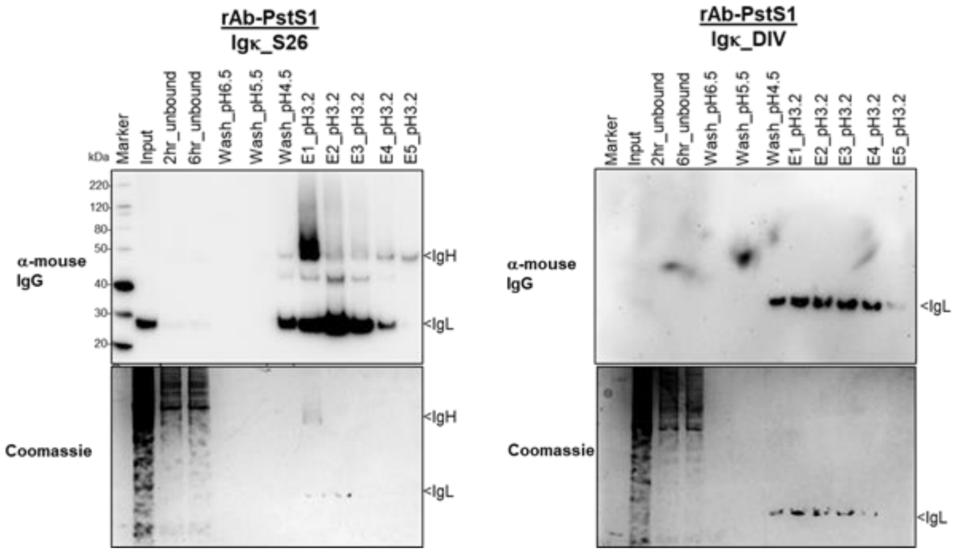

Original gel/blot pictures

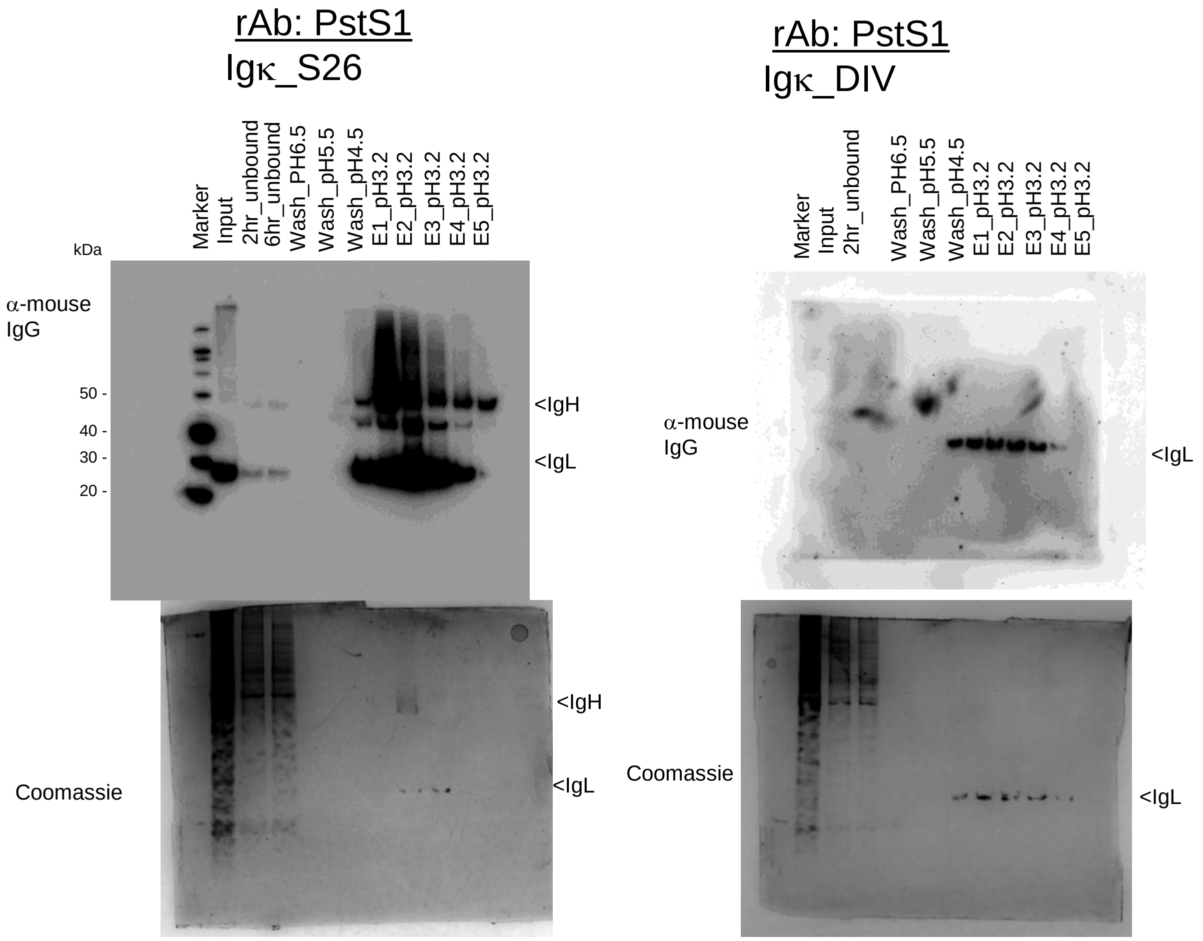

Top α–mouse IgG panel lists the blots developed by ECL and captured by Bio-Rad Image Lab 6.0 with adjustable brightness transformation. The image of the original blots is shown with higher saturated pixels than the final figures for an easy view of the gel boundary. Note that every image is from a whole, un-crop gel.

Final Figure 4s-B

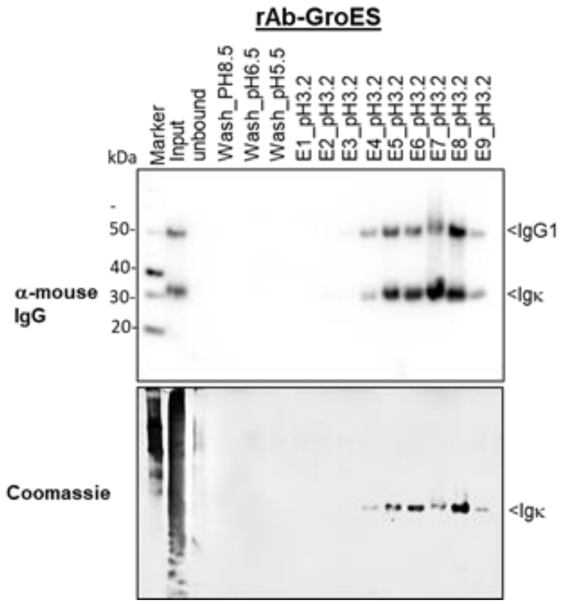

Original gel/blot pictures

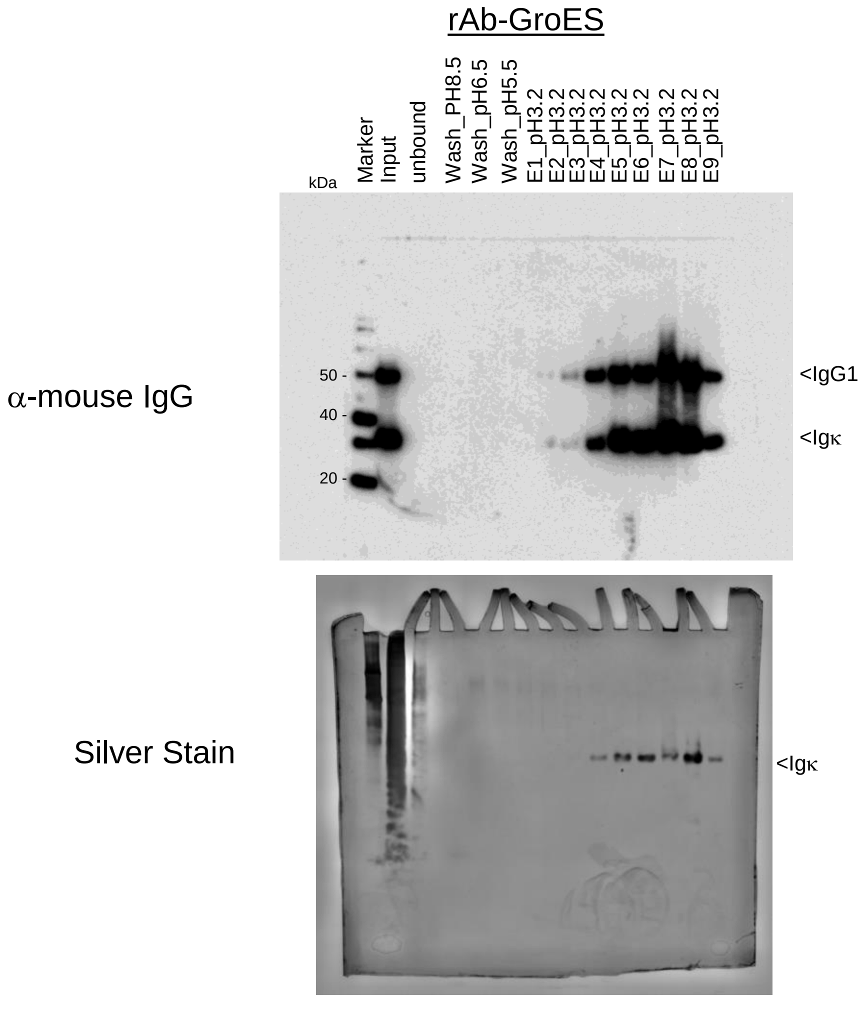

Top α–mouse IgG panel lists the blots developed by ECL and captured by Bio-Rad Image Lab 6.0 with adjustable brightness transformation. The image of the original blots is shown with higher saturated pixels than the final figures for an easy view of the gel boundary. Note that every image is from a whole, un-crop gel.

Final Fig 4s-C

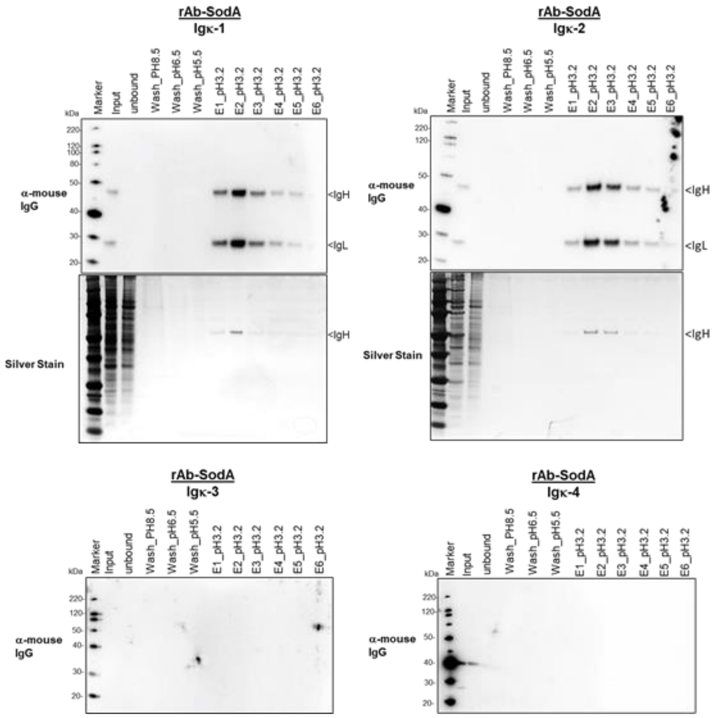

Original gel pictures

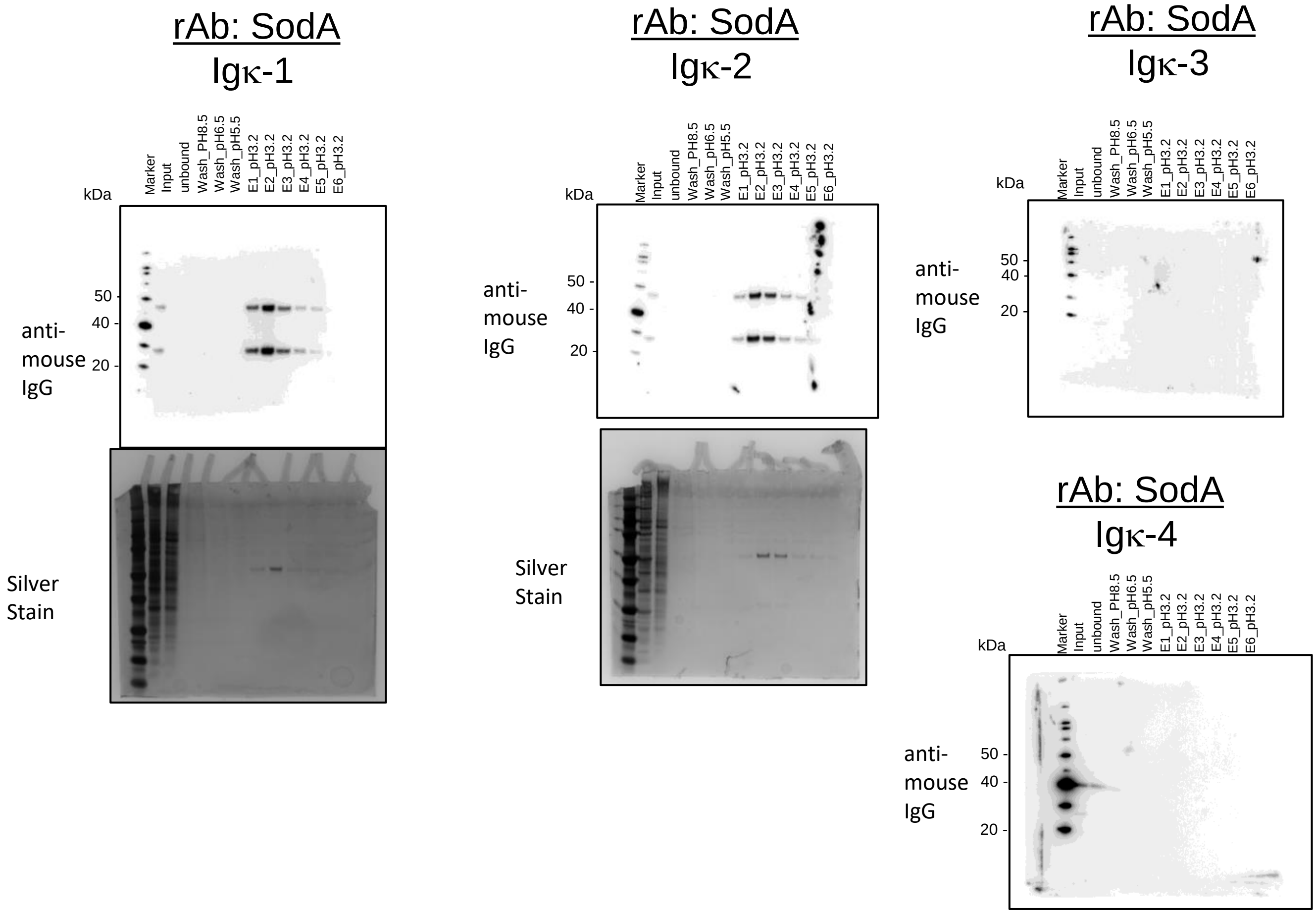

Final Figure 4s-D

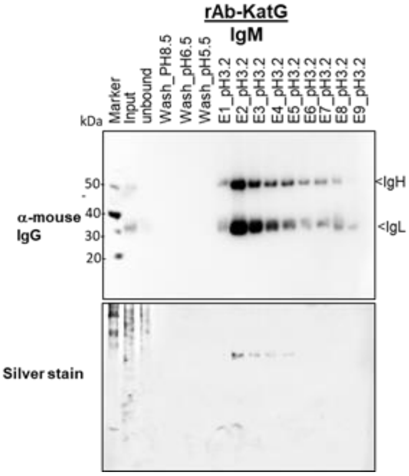

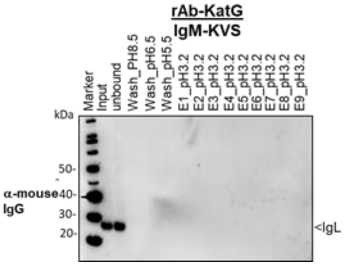

Original gel/blot pictures

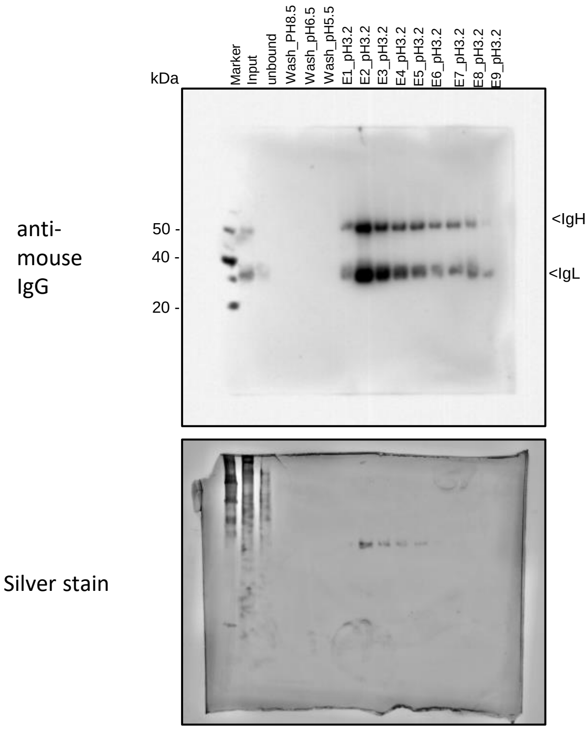

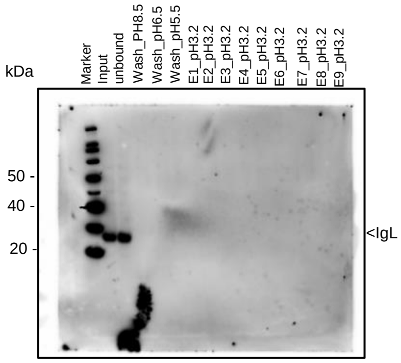

Top α–mouse IgG panel lists the blots developed by ECL and captured by Bio-Rad Image Lab 6.0 with adjustable brightness transformation. The image of the original blots is shown with higher saturated pixels than the final figures for an easy view of the gel boundary. Note that every image is from a whole, un-crop gel.

Final Figure 5s-A & B

A. rSodA

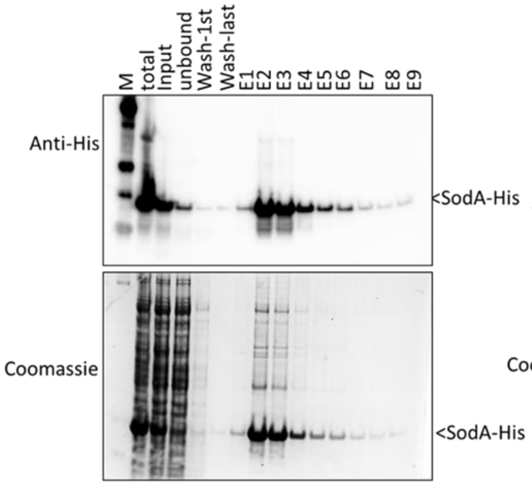

B. rKatG

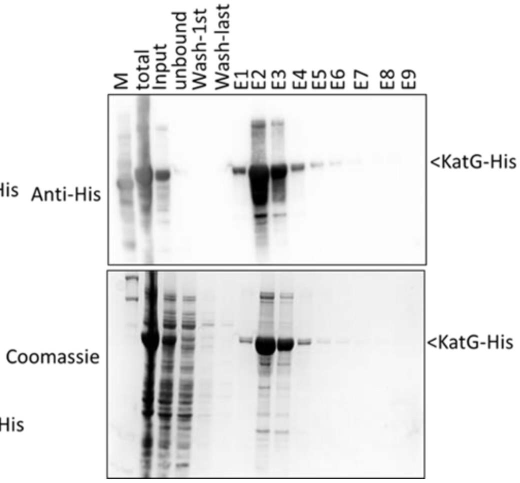

Original gel/blot pictures

A. rSodA

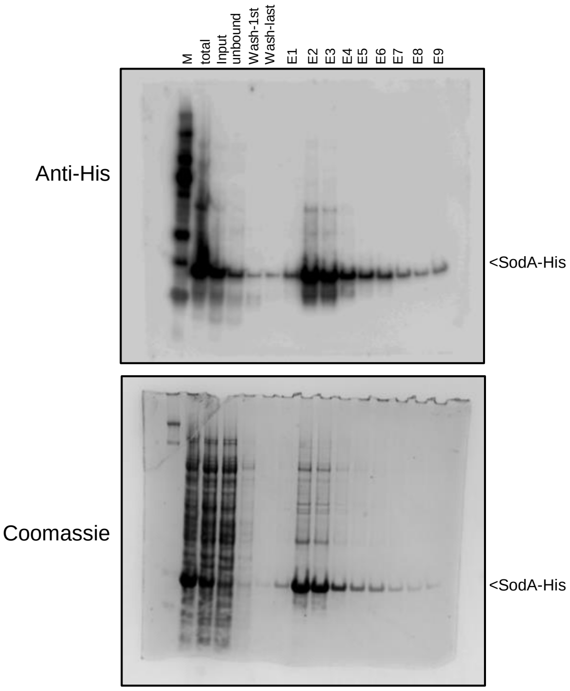

B. rKatG

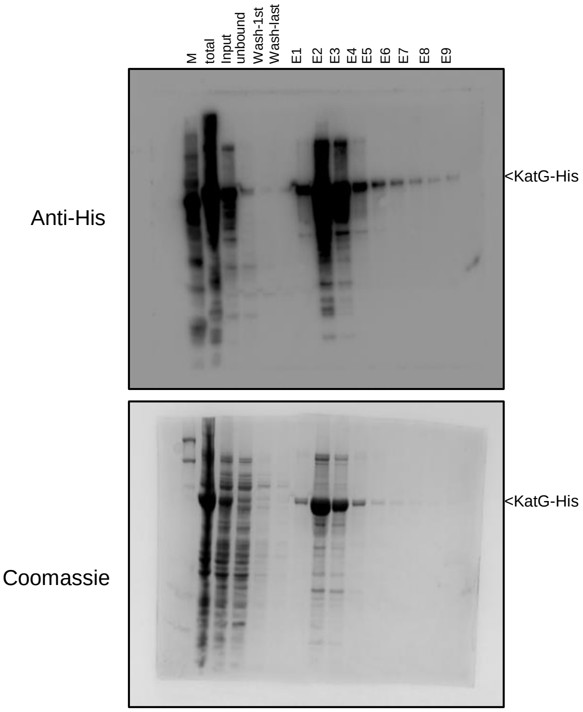

Top α–mouse IgG panel lists the blots developed by ECL and captured by Bio-Rad Image Lab 6.0 with adjustable brightness transformation. The image of the original blots is shown with higher saturated pixels than the final figures for an easy view of the gel boundary. Note that every image is from a whole, un-crop gel.
