# Supplementary material for: Determination of variable region sequences from hybridoma immunoglobulins that target Mycobacterium tuberculosis virulence factors
Source: PLoS One. 2021 Aug 20;16(8):e0256079. doi: 10.1371/journal.pone.0256079 (PMC8378720; doi:10.1371/journal.pone.0256079)
Supplement: Attachment — Submitted filename: Rebuttal_PONE-D-21-15036.docx [file pone.0256079.s019.docx]

PONE-D-21-15036
Determination of variable region sequences from hybridoma immunoglobulins that target Mycobacterium tuberculosis virulence factors
PLOS ONE

> We want to express our thanks to both reviewers and to the editor for the encouraging feedback and the opportunity to revise our manuscript. We are grateful for the thorough and careful review. We have addressed the concerns in a point-by-point response below. This thoughtful review process has improved the revised manuscript, and we hope you find it worthy for publication. Please note that all manuscript line number changes addressed below refer to text visible when “No Markup” is selected in the “Track changes” tab of Microsoft Word.

1. Please ensure that your manuscript meets PLOS ONE's style requirements, including those for file naming. The PLOS ONE style templates can be found at

<https://journals.plos.org/plosone/s/file?id=wjVg/PLOSOne_formatting_sample_main_body.pdf>

and

<https://journals.plos.org/plosone/s/file?id=ba62/PLOSOne_formatting_sample_title_authors_affiliations.pdf>

> *Our manuscript meets PLOS ONE’s style requirements, including those for file naming.*

2. PLOS ONE now requires that authors provide the original uncropped and unadjusted images underlying all blot or gel results reported in a submission’s figures or Supporting Information files. This policy and the journal’s other requirements for blot/gel reporting and figure preparation are described in detail at <https://journals.plos.org/plosone/s/figures#loc-blot-and-gel-reporting-requirements> and <https://journals.plos.org/plosone/s/figures#loc-preparing-figures-from-image-files>. When you submit your revised manuscript, please ensure that your figures adhere fully to these guidelines and provide the original underlying images for all blot or gel data reported in your submission. See the following link for instructions on providing the original image data: <https://journals.plos.org/plosone/s/figures#loc-original-images-for-blots-and-gels>.

In your cover letter, please note whether your blot/gel image data are in Supporting Information or posted at a public data repository, provide the repository URL if relevant, and provide specific details as to which raw blot/gel images, if any, are not available. Email us at [plosone@plos.org](mailto:plosone@plos.org) if you have any questions

> *The original blot/gel image data are now provided in Supporting Information.*

3. We note that you have included the phrase “data not shown” in your manuscript. Unfortunately, this does not meet our data sharing requirements. PLOS does not permit references to inaccessible data. We require that authors provide all relevant data within the paper, Supporting Information files, or in an acceptable, public repository. Please add a citation to support this phrase or upload the data that corresponds with these findings to a stable repository (such as Figshare or Dryad) and provide and URLs, DOIs, or accession numbers that may be used to access these data. Or, if the data are not a core part of the research being presented in your study, we ask that you remove the phrase that refers to these data.

> *The statement “Recombinant KatG was unstable in this strain as a His-tag antibody detected multiple degradation products (data not shown)” has been removed.*

Reply to the Reviewer #1 (Dr. Sinha)

1. It is stated (with the help of Figures 3 and 4) that the epitope binding potency and specificity of IgV paratopes were unaffected by Fc-isotype switch. Even so, the concern remains whether both antibodies (rAb and hyAb) are binding to the same epitope. One way to address this is to allow one of them to compete with the other during immunoblotting and ELISA.

> *Although rAb and hyAb exhibit similar affinity and specificity in the antigen recognition, direct analysis of the interface between paratope and epitope has not been performed.* Therefore, we changed the wording of “epitope-binding” to “antigen-binding” throughout the text. *We agree that Figure 3 and 4 only demonstrate the antigen-binding potency of rAb and hyAb. When biochemical features such as affinity and specificity are similar (Figs 3C-3D and Figs 4B-C), the underlying molecular mechanisms (e.g. paratope:epitope interaction), are often conserved.*

*However, to address Dr. Sinha’s question on measuring the epitope-binding potential between rAb and hyAb, we chose PstS1-hyAb and its rAb as an exemplar. Among the 4 hybridoma clones, the PstS1 clone elicited the most notable, differential affinity between rAb and hyAb. We examined if rAb and hyAb of PstS1 can compete for PstS1 in a direct competitive iELISA assay (****Fig 8s****). With PstS1 antigen as the limiting reagent (****Fig 8s****, blue), PstS1-hyAb, a lower affinity antibody than rAb, can still outcompete PstS1-rAb in PstS1-binding, eliminating almost all PstS1-rAb binding, clearly indicating that both PstS1-rAb and -hyAb recognize co-localized or identical PstS1 epitopes. This result supports our hypothesis that PstS1 hybridoma is polyclonal, secreting a mixture of antibodies with varied affinities.*

*We have added the antibody competition result in the Result section from line 302 to line 305 in the edited text, and description of the method in the Materials and Methods section from line #573 to line #579 of the edited text.*

2. As the authors have stated, glycosylation at specific sites plays a very important role in overall function of the antibody. Hence, they have coexpressed the IgVH and IgVL constructs in 293F cells. However, have they also checked whether the glycosylation actually happened in the rAb and whether it was equal or equivalent to that seen in hyAb?

> *As the reviewer mentioned, glycosylation is very important for antibody function. The majority of glycan studies center on the glycosylation impact on Fc-mediated antibody effector functions; however, a few studies mention glycan impact on Ab stability and recognition. Though our focus lies in the paratope determining IgV sequences, and not the Fc-region, expression of rAbs in 293F with post-translation modifications mimicking that of mammalian B cells avoids possible issues such as Ab aggregation, secretability, and/or alterations in antigen-binding. As demonstrated in Figs 3c and 3d, rAb^GroES^ expressed in 293F cells featured comparable affinity and specificity toward Mtb-GroES as hyAb^GroES^.*

*We did not compare the glycan profiles between the rAb and hyAb, since the validation of rAb^GroES^ supports that our expression system preserves enough antibody features for interrogating IgV-determining paratope properties. However, we agree that knowledge of the glycan profiles between rAb and hyAb could benefit future Ab production for commercialization. However, glycan profiling will require more than simple sugar staining. Genetic approaches coupled with Mass Spectromery analysis may be necessary and lie outside the scope of this current paper.*

3. The CDRs and FRs for each validated IgV sequence were defined by bioinformatics, using KABAT/IMGT algorithms. However, not all the positions in the traditionally defined CDRs are important for binding. Many positions that contribute critically to the binding energy may reside outside of the CDRs. Moreover, different CDR identification methods may often identify radically different stretches. This and other limitations of the work may be stated in a paragraph under Discussion.

> *The reviewer’s points are well-taken. We have added caution statements about interpreting CDR/FR stretches in line #318 to #323 of the Result section under “Determination of CDRs and FRs from validated IgV sequences”. The application of CDR/FR information was added in line #396 to #404 of the Discussion section.*

4. It will be helpful if all bioinformatic tools used in this study are briefly described in the ‘Materials and Methods’ section.

> *We have provided additional description of the tools and modified the Materials and Methods, lines #455-462, as well as included our custom R-scripts in Appendix 3s*.

5. More details on Immunoblotting will be helpful for the reader.

> *We have added immunoblotting details in the Materials and Methods, line #555 to line #561.*

Reply to the Reviewer #2

1. Table 3: The domain sequences would read clearer if the CDR and FR sequences are expressed in amino acid sequence rather than gene sequence. The gene family for the VDJ used should also be detailed.

> *We have correspondingly changed Table 3 and added Table 6s to annotate the gene family-associated Kabat alignments to define CDR/FR stretches.*

2. The expression in Line 341: please rephrase ready detection.

> *We have rephrased the statement to “The heterogeneity of host immune responses to Mtb infection manifests in diverse clinical presentations, posing challenges for rapid diagnosis and treatment of TB.”*

3. As the authors mentioned that deep sequencing capability is preferred to uncover even less abundant Ig-encoding transcripts, was gene analysis or VDJ gene analysis done to identify the clonality of the hybridoma?

> *Thank you for pointing out that the NGS workflow could be extended to investigate hybridoma clonality. NGS offers high throughput, high fidelity and deep sequencing capability, perfect for sequence annotation of VDJ. Deciphering the molecular mechanisms driving genome heterogeneity, of course, is very interesting and could be beneficial for the study of B-cell derived tumors. However, we focus on deciphering the hybridoma’s IgV-transcript sequences responsible for encoding the functional, antigen-binding antibody. The source of aberrant chains and/or the mechanisms giving rise to aberrant chains or multi-clonality lies outside the scope of the current effort.*

4. Line 450 to 452: Finally, a custom R script was applied to both the assembled NGS contigs and TOPO cloning/Sanger sequencing datasets to identify the aberrant sequences shared among most hybridoma cell clones. Is the script provided?

> *The script is now provided in Appendix 3s.*

5. Line 363, Since IgV sequence identification relies on eliminating these aberrant chains, we created a reference library of aberrant chain sequences for in silico subtraction. How was this library characterized and validated? There is no mention of the details to the reference library in terms of the selection process, characterization, and validation.

> *The reference library of aberrrant chain sequences was built from a large IgV- Mtb hybridoma sequencing project. All hybridoma clones were made from fusion of* *SP2/0 myeloma cells. The 4 clones described in this report were members of that project. All hybridoma cell lines were sequenced using both TOPO and NGS/Bioinformatic methods. The shared IgV contigs between hybridoma clones were collected as common aberrant chain sequences, since each hybridoma clone was known to secrete antibodies recognizing distinct Mtb antigens. The known IgV aberrant chains collected from the published literature were used to populate the the aberrant chain reference library, described in lines #476-479. After filtration, none of the reported IgV sequences share sequence identity with the members of our aberrant library. We have added additional details to describe how we generated the aberrant library in lines #473-474.*

6. There is the added point that the sequence information provided would also help to improve structural studies especially those on antibody-antigen interactions which should be added in the discussion to highlight the importance of the study.

> *Thank you for the suggestions. We have added these points in Discussion lines #396-404.*

7. In addition, please provide a percentage of coverage when using Sanger vs NGS. Also discuss the potential reasons/complications that resulted in a lower coverage using Sanger.

> *The definition of coverage, also called depth of sequencing, is the number of reads aligned to a particular base in a target sequence, typically averaged across all bases in a given target sequence. The necessary coverage for NGS to uncover a TRUE IgV is hard to predict, since it depends on the expression level of the target transcript and the variable size of the IgV transcriptome arising from a mixture of true and aberrant chains. In other studies, coverages from 10X to 30X is sufficient to study human mutations or SNPs. Our sequencing depth reached greater than 50X. The paratope-determining IgV in each clone was successfully identified in this report, indicating that 50X coverage is sufficient depth for hybridoma IgV sequencing.*

*> The sequencing principles of Sanger and NGS are different. NGS employs a parallel sequencing methodology, whereas Sanger sequencing is a clonal sequencing method. Once a sequencing clone is selected, Sanger sequencing is capable of producing one forward read and one reverse read, which depending on read overlap, can only produce between 1-2X coverage (considering potential strand read discrepancies). The advantage of the Sanger method resides in its longer, continuous reads, as noted in lines #166-170, which typically can reach up to 1kb. In our IgV amplicon, averaging around 500-600 bp, the IgV sequence was determined using a forward and a reverse primer. Thus, all IgVs reported here using the Sanger method have 2X coverage. Detection sensitivity of TRUE IgV lies in the initial selection of TOPO clones. Herein, we randomly selected 7-10 clones in each TOPO-IgV cloning. As shown in Table 2, many failed to uncover a true IgV sequence. Clearly, NGS provided a much more robust and greater sensitivity of detection, as previously described in lines #205-207.* *We have modified/added lines #354-361 in Discussion to emphasize the superiority of the NGS method in uncovering TRUE IgV.*

8. The apparent V,D,J segments used and their combination should be mentioned and discussed. Also, are the combinations commonly used in TB

> *As described in line #122 and* ***Fig 1A****, our PCR approach to isolate an IgV amplicon employed a 5’-universial prime and a gene (isotype) specific primer (ISP). The ISP aligned with the conserved sequence of CH, Constant region of Ig chain (****Table 1 & 1s****) so that multiple primers aligned with V-D-J gene segments were unnecessary for amplification. We avoid a multiplex PCR method which frequently gives rise to a distorted representation of immune repertoire due to primer bias. No segments or combinations were used, so no discussion is possible.*

*>* *Mycobacterial antigens trigger a diverse array of B cells-VDJ recombination. Heavy chains of IgM, IgA, IgE, IgG1, IgG2, IgG3 isotypes/subclasses in combination with either light chains of kappa or lambda can be found in human TB patients. In mice, Mtb-antigens induce similar humoral profiles consisting of IgM, IgA, IgG1, IgG2a, IgG3, kappa and lambda. Nevertheless, we cannot comment on combinations in the manuscript since we did not employ them.*

9. There are some spelling and grammatical errors which would benefit from another round of editing.

> *Thank you. We have made corrections in Tables (fonts) and in the text throughout using tracked changes in Word. We also further confirm our format to PLOS requirements.*

*> Please note we have also rearranged the order of presentation of* ***Figures 4A*** *to* ***4C*** *in the revised Results section.*

*> We have made corrections in the following Tables/Figures*

***Table 3****: We have changed the data from nucleic acid to amino acid presentation and added a footnote indicating the source of gene family used for the alignment (Table 6s)*

***Fig 1A****. Correction on IgL-mRNA label: change V_H_ to V_L_*

***Fig 4B****. Correction on Immunoblotting/Mw Labels: 50- to 40- & alignments*

***Table 6s****: Added Table.*

***Fig 1s-B.*** *Reformatted the A_260_/A_280_ label*

***Fig 4s-B****. Correction on bottom label: Coomassie to Sliver Stain*

***Fig 6s****.* *Reformatted the NRC-2894_Igκ label*

***Fig 8s****. Added Figure.*

***Appendix 3s****: Added Appendix*
