# Supplementary material for: Determination of variable region sequences from hybridoma immunoglobulins that target Mycobacterium tuberculosis virulence factors
Source: PLoS One. 2021 Aug 20;16(8):e0256079. doi: 10.1371/journal.pone.0256079 (PMC8378720; doi:10.1371/journal.pone.0256079)
Supplement: Attachment — Submitted filename: 2nd_Rebuttal letter.docx [file pone.0256079.s020.docx]

PONE-D-21-15036R1

Determination of variable region sequences from hybridoma immunoglobulins that target Mycobacterium tuberculosis virulence factors

PLOS ONE

> We have addressed the concerns in a point-by-point response below and we hope you find it worthy for publication. Please note that all manuscript line number changes addressed below refer to text visible when “No Markup” is selected in the “Track changes” tab of Microsoft Word.

PLOS ONE

Journal Requirements:

1. Please review your reference list to ensure that it is complete and correct. If you have cited papers that have been retracted, please include the rationale for doing so in the manuscript text, or remove these references and replace them with relevant current references. Any changes to the reference list should be mentioned in the rebuttal letter that accompanies your revised manuscript. If you need to cite a retracted article, indicate the article’s retracted status in the References list and also include a citation and full reference for the retraction notice.

> We have reviewed our reference for completeness and accuracy.

1. In manuscript line #340, we removed the reference #71 and replaced it with references #4 & #5. (Reference #71 was not the correct reference)

2. We also check if our references meet PLOS journal format recommendations (<https://journals.plos.org/plosone/s/submission-guidelines>). We have made the following changes.

2.1. Ref #4: added a Web link

2.2. Ref #5: corrected author information and conformed to journal citation format

2.3. Ref #18: font size adjustment

2.4. Ref #55: conformed to journal citation format

2.5. Ref #67: conformed to journal citation format

2.6. Ref #68: conformed to journal citation format

2.7. Ref #70: conformed to journal citation format

2.8. Ref #71: removed. It is an incorrect reference.

Review Comments to the Author

Reviewer #1: I am satisfied with authors’ response to my queries/comments but it would be nice if they could address one lingering concern about the data shown as Fig 8S. A large amount of PstS1 hyAb was needed to inhibit the binding of rAb, which raises concern about the specificity of this competitive binding. Could it have arisen from a non-specific mechanism such as steric hindrance? Is it possible to address this doubt with the use of an ‘isotype control’ (same IgG subclass but with no or irrelevant antibody activity)?

> Thank you for the suggestion. As we have mentioned in our 1^st^ Rebuttal letter, we have changed the wording from epitope-binding to antigen-binding throughout the text since we did not yet provide a direct analysis on the interface between paratope and epitope in Figs 3 & 4. We performed a competitive assay with isotype controls to address your concern over non-specific competition. In Fig 8S, the competitive assay, we tested whether rAb and hyAb of the same antigen can compete for their recognition epitopes. PstS1 group is of particular interest since the hyAb seems to be around 80% as potent as its rAb. In the previous Fig 8S, we did a quick diagnosis, and it was very promising. To provide a more stringent and quantitative analysis, we have revised our Fig 8S and made the modifications listed below.

1. We decreased the amount of PstS1 antigen from 2 to 1 ug/ml in the overnight coating to the ELISA plate. The reduced number of epitopes should create more selective binding competition between rAb and hyAb, avoiding/reducing the amount of non-specific binding or Ab absorption.

2. All antibody and antigen reagents used herein are purified or purchased in purified form, thereby reducing the likelihood of impurities interfering with the readouts.

3. In addition, we also cross-examined all the antibody reagents used herein for their recognition specificity (**Fig 8sB**).

4. We also included an hAb’s isotype control in parallel to control for non-specific mechanisms at high concentrations of antibody. hyAb-SodA, an IgG1κ isotype, has been purified to homogeneity and is highly selective for its binding antigen (**Fig8sB**, blue dash). As shown in **Fig 8sA**, hyAb-PstS1 inhibits rAb-PstS1 binding in a dose-dependent manner, whereas its isotype control, hyAb-SodA, cannot, indicating that the assay is highly selective and depends on specific antibody recognition of the antigenic sites (epitopes).

5. Quantitatively, our assay is in good agreement with the relative affinities between rAb and hyAb-PstS1 (**Fig 4A**, similar, within 20% difference). Shown below is the Ag-Ab binding equation:

- When Ka_1_ is relatively close to Ka_2_ (less than 1 log difference; in our scenario, less than 20% difference), Ag-Ab complexes comprise roughly a 1:1 ratio of Ab_1_- and Ab_2_-components when Ab_1_ and Ab_2_ have equimolar starting concentrations. We observed that the concentration of hyAb-PstS1 antibody required to achieve 50% inhibition is between 64 and 128 ng/ml, quite comparable to the concentration of rAb-PstS1 in the reaction (100 ng/ml).
- Only by increasing the relative concentration of one antibody over the other (significantly) can one achieve “complete” inhibition of the other’s binding when both Ka_1_ and Ka_2_ are similar. 1 log (herein 1ug/ml) higher concentration of hyAb over that of rAb should begin to strongly favor hyAb binding, consistent with our data.

> We modified lines #302-309 and #577-585 to reflect this result.
